# Intestinal inflammation induces glymphatic remodeling, priming early neurodegenerative signals in male mice

**DOI:** 10.1002/alz.70640

**Published:** 2025-10-09

**Authors:** Clara Ciampi, Francesca Fagiani, Valentina Murtaj, Federica Comella, Veronica Torre, Marta Filibian, Annapaola Andolfo, Clarissa Braccia, Nicola Opallo, Maria Grazia Bottone, Edoardo Pedrini, Elena Lucarini, Maria Bove, Stefano Govoni, Carla Ghelardini, Rosaria Meli, Luigia Trabace, Anna Pittaluga, Giuseppina Mattace Raso, Lorenzo Di Cesare Mannelli, Cristina Lanni

**Affiliations:** ^1^ Department of Neuroscience Psychology Drug Research and Child Health‐NEUROFARBA Pharmacology and Toxicology Section University of Florence Florence Italy; ^2^ Department of Drug Sciences University of Pavia Pavia Italy; ^3^ Division of Neuroscience Vita‐Salute San Raffaele University Milan Italy; ^4^ Department of Pharmacy School of Medicine University of Naples “Federico II,” Naples Italy; ^5^ Department of Pharmacy University of Genoa Genoa Italy; ^6^ Centro Grandi Strumenti University of Pavia Pavia Italy; ^7^ INFN, Istituto Nazionale di Fisica Nucleare‐Pavia Unit Pavia Italy; ^8^ ProMeFa Proteomics and Metabolomics Facility IRCCS San Raffaele Scientific Institute Milan Italy; ^9^ Department of Biology and Biotechnology Laboratory of Cellular Biology and Neurobiology University of Pavia Pavia Italy; ^10^ Center for Omic Sciences Vita‐Salute San Raffaele University Milan Italy; ^11^ Department of Clinical and Experimental Medicine University of Foggia Foggia Italy; ^12^ Centro 3R (Inter‐University Center for the Promotion of the 3Rs Principles in Teaching and Research) Pisa Italy

**Keywords:** aquaporin‐4, brain waste clearance, circadian regulation, dextran sulfate sodium–driven painful colitis, inflammation, neurotransmitter release

## Abstract

**INTRODUCTION:**

Inflammatory bowel disease triggers extraintestinal manifestations, including in the central nervous system (CNS). However, the direct impact of peripheral inflammation on the CNS is largely unknown.

**METHODS:**

Using a mouse model of colitis with pain and anxiety‐like behavior, we investigated the intricate pathogenic link between colonic inflammation, disruptions in circadian rhythmicity and impaired glymphatic circulation.

**RESULTS:**

By in vivo magnetic resonance imaging, we observed a derangement of brain fluid dynamics, with a significant enlargement of the cerebral lateral ventricles and waste deposition within the brain parenchyma. Proteomics revealed changes in cerebrospinal fluid (CSF) composition, enriched in proteins related to inflammation, immune response, complement, neuronal, and lipid metabolic pathways. Alterations in brain metabolite concentrations and in inhibitory control mechanisms and excitatory transmission were detected.

**DISCUSSION:**

Colonic inflammation induces remodeling in CSF volume distribution, clearance, and metabolism, with derangement of the crosstalk between neurons and astrocytes, priming synaptopathy.

**HIGHLIGHTS:**

- An acute peripheral inflammatory trigger affects the central level by remodeling central nervous system (CNS) fluid distribution and priming early signals of synaptopathy.
- A single dextran sulfate sodium (DSS) challenge disrupts the circadian clock machinery and alters CNS fluid distribution, a so far neglected system, thereby impairing glymphatic clearance of waste products and indirectly altering neurotransmitter release dynamics.
- These combined effects ultimately impact brain function, extending to the regulation of behavior.
- Understanding how an intestinal inflammatory insult may derange the daily rhythm of the mechanisms controlling brain waste disposal may help identify specific groups of subjects at high risk of developing neurological disorders.

## 1 BACKGROUND

A strong link between colonic inflammation and central nervous system (CNS) alterations, leading to synaptic derangements, has been suggested. Such connection is primarily mediated through the gut–brain axis, a complex network of communication pathways between the gastrointestinal tract and CNS. Several studies have demonstrated a correlation between inflammatory bowel diseases (IBD), such as Crohn's disease (CD) and ulcerative colitis (UC), and higher rates of depression and anxiety.^1^ Recent meta‐analyses revealed a higher risk of neurodegenerative disorders, such as Alzheimer's disease (AD) and Parkinson's disease (PD), among patients affected by CD and UC compared to the general population.^2^, ^3^ The mechanisms underlying the connection between colonic inflammation and neurodegeneration and age‐related disorders are still being investigated. Peng et al.^2^ highlights that the association between IBD and increased risk of AD may be confounded by other variables, possibly related to the effects of inflammation on various biological markers. This underscores the complexity of the relationship between peripheral inflammation and neurodegeneration and highlights the need for further investigation to fully grasp potential causal pathways. Despite the lack of definitive causation, these results strengthen the notion that individuals with IBD may face an elevated risk of developing dementia, thus justifying heightened clinical awareness and early intervention strategies in this patient population.

Neuroinflammation has been proposed as one of the main mechanisms linking gut dysfunction and dysbiosis to age‐related disorders.^4^ Chronic colonic inflammation leads to a systemic inflammatory response, associated with pro‐inflammatory cytokines’ release into the bloodstream, in turn crossing the blood–brain barrier (BBB) and contributing to neuroinflammation.^5^ Circulating pro‐inflammatory mediators in IBD patients have been shown to play a role in the development of several illnesses affecting the CNS.^6^ Furthermore, the gut is directly connected with the CNS by vagal, autonomic, and spinothalamic afferents. These pathways are sensitized in inflammatory conditions, a phenomenon contributing to the centralization of pain, as well as to its persistence, a disabling condition of IBD patients.^7^ Moreover, in animal models, a strong link between colitis and cognitive deficits, as well as changes in brain structure, has been reported.^5^, ^8^ Even if the identification of these potential risks may suggest earlier preventive measures to reduce future comorbidity, elucidating the mechanisms and the specific pathways involved is needed to fully understand the complex relationship between gut inflammation and brain (dys)function.

Evidence from literature suggests that the choroid plexus, responsible for the production of the cerebrospinal fluid (CSF), responds to peripheral inflammatory stimuli as a relevant transducer of signals between the periphery and the brain.^9^, ^10^ The glymphatic system is important in CNS fluid dynamics by regulating the exchange between CSF in perivascular spaces and interstitial fluid (ISF) within the parenchyma, facilitating solute distribution, and ultimately promoting drainage along lymphatic pathways.^11^ Notably, the glymphatic system is under circadian regulation,^12^ and its function is impaired in neurological diseases, including, but not limited to, neurodegenerative conditions.^13^ In physiological conditions, CSF flux and gut activity follow a finely regulated circadian rhythm, governed by internal biological clocks and external synchronizing signals.

Based on this evidence, we investigated whether peripheral colonic inflammation can disrupt the circadian clockwork not only in the periphery^14^ but also in the CNS, affecting its physiological functions, such as the regulation of brain fluid distribution and clearance. Here, through an acute model of dextran sulfate sodium (DSS)‐induced colitis in male mice, we detailed an impairment in brain fluid dynamics and clearance associated with a derangement of the crosstalk between neurons and astrocytes, thus priming signals of synaptopathy. DSS‐induced colitis is a widely used animal model for studying IBD. The acute form highlights innate immune involvement, while the chronic model, triggered by repeated DSS exposure, includes adaptive immunity, resulting in complex inflammation and lasting neuroinflammation.^15^ Our study highlights that a single DSS challenge disrupts the circadian clock and alters CNS fluid distribution, a so far neglected system, impairing glymphatic waste clearance and indirectly modifying neurotransmitter release dynamics.

## 2 METHODS

### 2.1 Study design

The following five experimental studies were performed. (1) Reproduction of the DSS‐induced colitis mouse model, recapitulating histological and molecular features and characterizing pain‐related behaviors and mood disturbances. (2) Clockwork machinery disruption in DSS, configured as a key player of brain fluid dysfunctions. (3) Functional assessment of brain fluid distribution impairment by magnetic resonance imaging (MRI). (4) Alterations in synaptic functionality driven by the loss of the homeostatic properties of the astrocyte‐mediated glymphatic system, leading to impaired neuron‐astrocyte crosstalk. (5) Analysis of brain metabolite concentrations in colitis conditions, as an index of brain tissue response. The animal sample size was calculated by performing a power analysis. Exact numbers for each experiment are included in the figure legends. Animals were used from different litters, an important step for randomization to reduce bias. The animals were randomly allocated to the different experimental groups and test batteries (see Figure S1 in supporting information). The effect of DSS was evident on animals, in line with the colitis‐like animal models, thus making it impossible to have completely blinded investigators. For behavioral batteries, investigators were blinded to group allocation during data collection and analysis.

### 2.2 Animals

**RESEARCH IN CONTEXT**

1. **Systematic review**: Growing evidence suggests that gastrointestinal disorders contribute to the onset of neuroinflammation and central comorbidities, such as anxiety, mood disorders, and somatic hypersensitivity. Epidemiological data indicate a higher predisposition among patients with chronic irritable bowel diseases (IBD), such as Crohn's disease and ulcerative colitis, to develop neurodegenerative conditions like Alzheimer's disease and Parkinson's disease. A hallmark of these neurodegenerative disorders is the disruption of circadian clockwork homeostasis, which is closely linked to the regulation of brain fluid dynamics.
2. **Interpretation**: Our data suggest that peripheral inflammation induced by colitis is mirrored at the central level, disrupting the circadian framework and leading to an imbalance in brain fluid dynamics and ventricular enlargement. This is particularly marked by impaired astrocytic function in glymphatic regulation, resulting both in altered crosstalk with neurons with consequent development of synaptopathy and in accumulation of phosphorylated tau and amyloid oligomers in brain parenchyma. These findings point to a potential mechanistic link between peripheral inflammation and increased susceptibility to neurodegenerative diseases.
3. **Future directions**: We suggest redirecting basic research toward understanding the mechanisms underlying recovery from acute colitis, while also characterizing the alterations in a model of chronic intestinal injury. Within this framework, the glymphatic system can be configured as a candidate target to direct pharmacological research on IBD, with the ultimate goal of preventing long‐term comorbidities.

Eight‐week‐old male C57BL/6N mice were obtained from ENVIGO RMS SRL (San Pietro al Natisone) and Charles River and bred inside the animal facility at the Interdepartmental Service Center for the Unified Management of Housing and Radiobiology Activities of the University of Pavia and the CeSAL (Centro Stabulazione Animali da Laboratorio, University of Florence, Florence, Italy). Four mice were housed per cage (size 26 × 41 cm) and used at least 1 week after their arrival. Mice were group‐housed in a 12:12 light/dark cycle and with ad libitum access to food and water. All the animal holding rooms were maintained at a temperature of 21 ± 1°C and humidity of 50% ± 5%. All animal care and experimental procedures were in compliance with guidelines approved by Ministerial Authorizations (number 146/2022‐PR, protocol n. B2EF8.28, number 105/2023‐PR, protocol n. 0028580, number 808/2021‐PR, protocol n. 17E9C.240, number 1046/2023‐PR, protocol n. 17E9C.284). All efforts were made to keep animal usage to a minimum, in accordance with the achievement of project aims. Our study examined male mice, based on previous literature on the same animal model.^16^, ^17^ The choice of male mice may sound like a limitation for the study, because some neurodegenerative disorders, like AD, show sex differences. However, the use of male mice is justified by the fact that colitis exhibits an overall equal prevalence between sexes.^18^ Moreover, female mice have been found to develop less severe colitis and hence are partially protected against chemically induced colitis. According to the literature, female mice would have needed at least the synchronization of the menstrual cycle, because the bowel inflammatory response depends upon circulating estrogen levels.^19^ Furthermore, concerning the age of animals used, the study is focused on identifying informative features present during the very early stages predisposing to neurodegenerative disorder, in line with the report published by the Lancet Commission on Dementia Prevention, Intervention, and Care,^20^ supporting the choice of young animals over older animals.

### 2.3 DSS‐induced acute colitis model

Acute colitis was induced in mice by administering 2.5% (w/v) DSS dissolved in drinking water for 5 days, followed by 72 hours of wash‐out. Control animals received tap water without DSS. The percentage of body weight loss was calculated starting from the second day of DSS treatment until the time of sacrifice. The severity of colitis was determined based on colon length differences between control and DSS‐treated animals, body weight loss, stool consistency, and bleeding. Weight, presence of blood, and gross stool consistency were determined daily in all mice. All these parameters were used to calculate the disease activity index (DAI), scored from day 1 pre exposure to 2 days post exposure to DSS. The DAI was calculated as follows: DAI = (weight loss score + fecal consistency + blood in the feces)/3. DAI scores were determined as follows: weight loss: 0 (no loss), 1 (1%–5%), 2 (5%–10%), 3 (10%–20%), and 4 (> 20%); stool consistency: 0 (normal), 2 (loose stool), and 4 (diarrhea); bleeding: 0 (no blood), 1 (hemoccult positive), 2 (hemoccult positive and visual pellet bleeding), and 4 (gross bleeding, blood around anus).

Animals were sacrificed by cervical dislocation on the eighth day, corresponding to the peak of the acute experimental setting. After sacrifice, brains, cerebral areas (hypothalamus, hippocampus, and cortex), and colons were collected for biochemical analyses. Colon length and weight were recorded.

### 2.4 Pain assessment

Viscero‐motor response (VMR) and abdominal withdrawal reflex (AWR) to colorectal distension (CRD) were performed according to a previously described method,^21^ recording electromyographic (EMG) signals and behavioral score, respectively. Thermal allodynia and hyperalgesia were assessed using the cold and hot plate tests, respectively. Pain‐related behavior (licking of the hind paw) was observed, and the time (seconds) of the first sign was recorded. The cutoff time of the latency of paw lifting or licking was set at 30 seconds.^22^ Mechanical allodynia was assessed using the Von Frey test. The paw sensitivity threshold was defined as the minimum pressure required to elicit a robust and immediate withdrawal reflex of the paw.^22^ Detailed experimental cohorts and procedures are provided in the supporting information.

### 2.5 Behavioral tests

To evaluate anxiety‐ and depressive‐like behaviors, together with cognitive impairments, we carried out two behavioral test batteries in two different cohorts of DSS‐induced acute colitis mice and relative controls. We performed such behavioral batteries by choosing the least invasive paradigms to minimize the impact on the behavioral response of the following tasks to reduce the number of animals used and, thus, accomplish the 3 Rs principles for animal research. In particular, we performed the following behavioral test batteries: (1) sucrose preference test, open field test, novel object recognition test, tail suspension test; (2) hole board test, splash test, elevated plus maze test, passive avoidance task.

**FIGURE 1:**
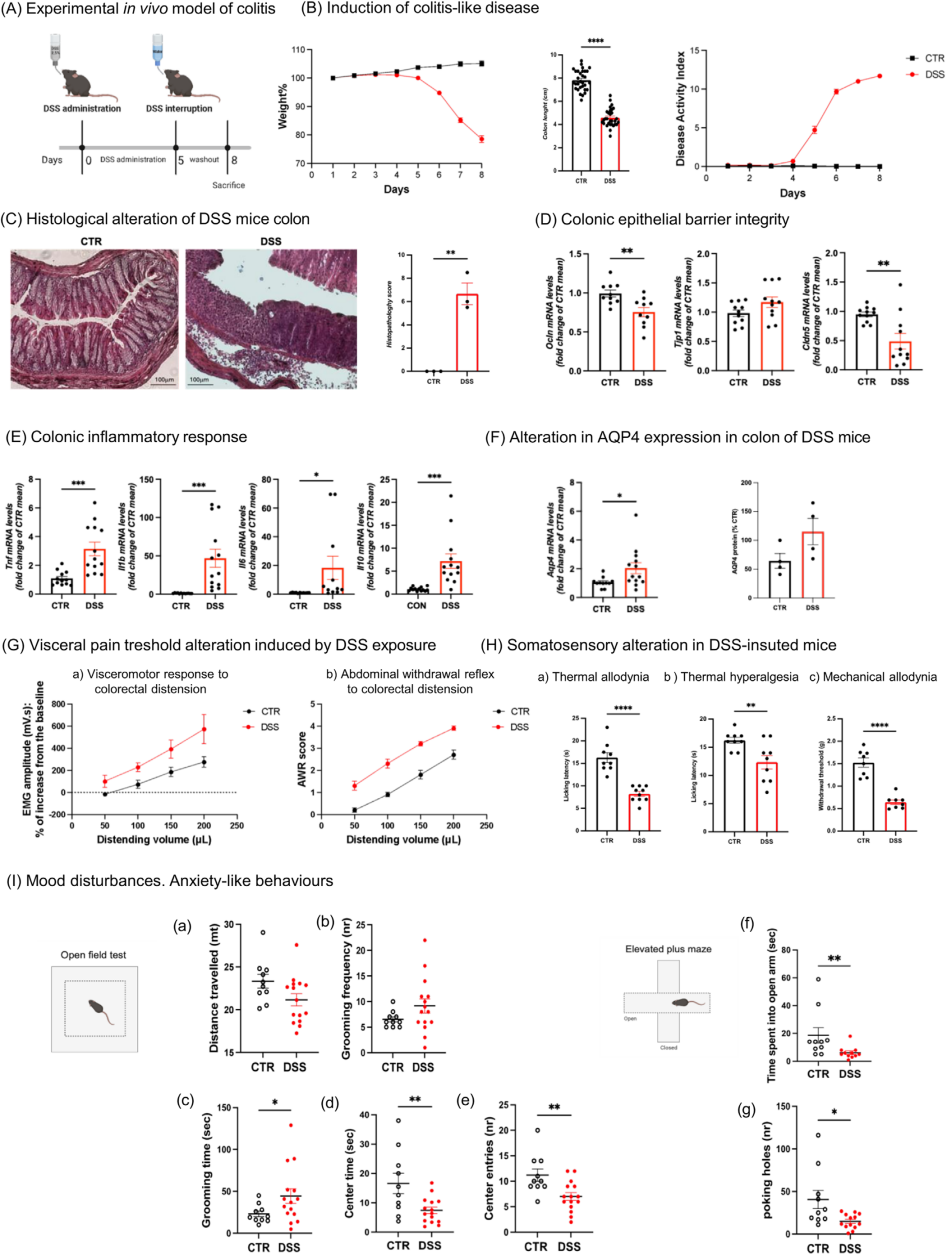
DSS‐induced colitis mouse model. A, Experimental setting for in vivo model of colitis: 2.5% DSS was dissolved in the drinking water and fed ad libitum to male C57BL/6N WT mice at 8 weeks of age for acute setting. B, The percentage of body weight loss was calculated starting from the second DSS treatment until the week of sacrifice. Data are expressed as mean ± SEM and analyzed using a two‐way ANOVA (condition and time were considered variables) with a Bonferroni post hoc test (****P < 0.0001). Colon length was measured after the sacrifice. Data are expressed as mean ± SEM and analyzed using a two‐tailed unpaired Student *t* test (****P < 0.0001). DAI was scored from day 1 pre exposure to 2 days post exposure. Weight, presence of blood, and gross stool consistency of all mice were determined daily. Data are expressed as mean ± SEM and analyzed using a two‐way ANOVA (condition and time were considered variables) with Bonferroni post hoc test (****P < 0.0001). C–F, Histological and molecular features of colonic tissue. C, Representative photomicrographs of H&E‐stained colon sections of CTR and DSS mice (scale bars represent 100 𝝁m) and histopathology scores. Data are reported as mean ± SD, *n* = 3/group. D, mRNA expression of the epithelial barrier junctions, Ocln (*n* = 10/group), Tjp1 (*n* = 11/group), and Cldn5 (*n* = 11/group), and (E) pro‐ and anti‐inflammatory cytokines, *Il1b* (*n* = 13/group), *Tnf* (CTR *n* = 12 and DSS *n* = 13), *Il6* (*n* = 11/group), and *Il10* (*n* = 13/group) was evaluated in colon tissues from CTR and DSS mice by rt‐PCR. Data are reported as mean ± SEM and analyzed using an unpaired two‐tailed *t* test (*P < 0.05, **P < 0.01, ***P < 0.001 vs. respective controls). F, mRNA (CTR *n* = 11 and DSS *n* = 13) and protein (*n* = 4/group) expression of Aqp4 in colon tissues. Data are reported as mean ± SEM and analyzed using an unpaired two‐tailed *t* test (*P < 0.05, **P < 0.01, ***P < 0.001 vs. respective control). G, H, Visceral and somatic pain. G, Visceral sensitivity was assessed by measuring the electromyography (EMG) amplitude of abdominal contraction VMR; (a) and scoring behavioral responses AWR; (b) in awake animals to colorectal distension with increasing volumes (50–200 µL balloon inflation). H, Somatic pain was assessed in mice by the cold plate test (thermal allodynia; (a) hot plate test (thermal hyperalgesia); (b) and the von Frey test (mechanical allodynia); (c) each value represents the mean  ±  SEM of nine animals per group (*n*  =  9). **P < 0.01 and ****P < 0.0001 versus controls and analyzed using a two‐way ANOVA with Šídák multiple comparisons test. Each value represents the mean  ±  SEM of 10 animals per group (*n*  =  10). *P < 0.05 and **P < 0.01 versus controls and analyzed using a two‐way ANOVA with Šídák multiple comparisons test. I, Anxiety‐like behavior: (a) distance traveled (in meters) in the open field test from control (CTR, black bar, *n* = 10) and DSS (red bar, *n* = 15) mice. Data are expressed as mean ± SEM. Two‐tailed unpaired Student *t* test, *t* = 1.979, df = 23, C.I. = −4.449 to 0.09815, η^2^ = 0.1455, *P* = 0.06 DSS versus CTR. b, Frequency of grooming behavior (in numbers) in the open field test from control (CTR, black bar, *n* = 10) and DSS (red bar, *n* = 15) mice. Data are expressed as mean ± SEM. Two‐tailed unpaired Student *t* test with Welch correction, *t* = 1.771, df = 17.36, C.I. = −0.4987 to 5.765, η^2^ = 0.1530, *P* = 0.0941 DSS versus CTR. c, Time spent performing grooming behavior (in seconds) in the open field test from control (CTR, black bar, *n* = 10) and DSS (red bar, *n* = 15) mice. Data are expressed as mean ± SEM. Two‐tailed unpaired Student *t* test with Welch correction, *t* = 2.278, df = 17.95, C.I. = 1.649 to 40.95, η^2^ = 0.2242, *P* = 0.0352; *P < 0.05 DSS versus CTR. d, Time spent in the center area (in seconds) of the open field arena from control (CTR, black bar, *n* = 10) and DSS (red bar, *n* = 15) mice. Data are expressed as mean ± SEM. Two‐tailed unpaired Student *t* test with Welch correction, *t* = 2.503, df = 10.92, C.I. = −17.30 to −1.103, η^2^ = 0.3645, *P* = 0.0295; *P < 0.05 DSS versus CTR. e, Numbers of entries in the center area of the open field arena from control (CTR, black bar, *n* = 10) and DSS (red bar, *n* = 15) mice. Data are expressed as mean ± SEM. Two‐tailed unpaired Student *t* test, *t* = 3.050, df = 23 C.I. = −7.048 to −1.352, η^2^ = 0.2880, *P* = 0.0057; **P < 0.01 DSS versus CTR. f, Time spent in the open arms (in seconds) of the elevated plus maze apparatus from control (CTR, black bar, *n* = 10) and DSS (red bar, *n* = 11) mice. Mann–Whitney test. Data are expressed as mean ± SEM. *P* = 0.0072; **P < 0.01 DSS versus CTR. g, Number of poking holes in the hole board task from control (CTR, black bar, *n* = 10) and DSS (red bar, *n* = 13) mice. Data are expressed as mean ± SEM. Two‐tailed unpaired Student *t* test with Welch correction, *t* = 2.333, df = 9.920, C.I. = −50.08 to −1.123, η^2^ = 0.3543, *P* = 0.0420; *P < 0.05 DSS versus CTR. ANOVA, analysis of variance; AWR, abdominal withdrawal reflex; CTR, control; DAI, disease activity index; DSS, dextran sulfate sodium; rt‐PCR, reverse transcription polymerase chain reaction; SD, standard deviation; SEM, standard error of the mean; VMR, viscero‐motor response; WT, wild type

The specifications for the behavioral tests included in Figure 1 are reported below. The other behavioral tests and animal cohorts were reported in detail in the supporting information.

On the seventh day, the open field test was performed to assess locomotor activity and anxiety‐like behaviors. Animals were acclimatized for 1 hour, then they were placed in a square, dark arena, under bright light, and let free to explore for 5 minutes. Video tracking software (ANY‐maze 7.01 version, Ugo Basile) recorded the test and automatically scored the distance traveled (in meters), the time spent in the center (in seconds), the number of entries in the center area, and the immobility time (in seconds), whereas a blind experimenter manually scored the duration (in seconds) and the frequency (in numbers) of grooming behavior.

On day 8, mice underwent the elevated plus maze task. The test was performed according previous protocol.^23^ Briefly, the elevated plus maze apparatus consisted of two opposite closed arms (with side walls), two opposite open arms (without side walls), and a central area. The mice were placed in the center area facing an open arm and let free to explore the maze for 5 minutes under a bright light. The time spent in the open arms (in seconds), considered a measure of anxiety‐like behavior in terms of an inverse correlation, was then scored by a blind experimenter.

### 2.6 Rhythmic gene expression analysis

Mice were euthanized at zeitgeber time (ZT) 0, 4, 8, 12, 16, and 20. In our experiments, the zeitgeber, an external cue helping to synchronize the internal biological clock with the environment, is represented by the light (daily cycle of light and darkness). The colon tissues and brain areas (hypothalamus, hippocampus, and cortex) were used in quantitative polymerase chain reaction (qPCR) to detect rhythmic signals from our time‐series gene expression data. The cDNA from colon and brain area tissues was analyzed for the gene expression profiles of the positive regulators (*Clock*, *Bmal1*), the negative regulators (*Per1*, *Per2*, *Cry1*, *Cry2*), and the transcriptional repressor *Nr1d1* within the circadian clock. *Gapdh* transcript was used as a reference control. The function meta2d, within the MetaCycle R package (PMC5079475), was used to estimate the phase and period of the expression signal. Finally, following the tool's recommendation (https://cran.r‐project.org/web/packages/MetaCycle/vignettes/implementation.html), we fit a linear model per gene and condition, defined as value ∼ trend+amplitude, where trend is defined as time_points—sum(time_points)/nrow(.) and amplitude is defined as cos(2*pi*(time_point‐phase)/period). The model was then used to exhibit the expression values for different genes/conditions at shorter time points. At least three animals per group were used for each time point. The primers used for the analyses were provided by Qiagen and are reported as supporting information data (extended data table primers).

### 2.7 Magnetic Resonance Imaging and Spectroscopy analysis

For a detailed description of animal preparation for in vivo imaging and MRI/magnetic resonance spectroscopy (MRS) acquisition protocol, see the supporting information.

Gadolinium (Gd) enhancement quantification (Gd‐T1 weighted MRI) was performed using PMOD 4.1 software. Briefly, for each animal (*n* = 12 controls: *n* = 14 DSS), and for each time point post contrast (TPC) injection (10 time points), images were manually co‐registered to an MRI template and the mean signal intensity (S(TPC)) extrapolated in several regions of interest (ROIs) automatically positioned on co‐registered images, resorting to a mouse brain atlas.^24^ The same procedure was applied to obtain the mean signal intensity in the ROIs positioned over the baseline images (Sb; *n* = 3 controls, *n* = 1 DSS), acquired without Gd injection. For each time point the percentage signal enhancement (SE [%]) ratio to baseline was obtained according to the expression SE = (S(TPC)/Sb)*100. An intensity enhancement analysis was conducted on 3D Slicer on a manual selected ROI based on the hyperintense areas marking the presence of Gd‐based contrast agent. This area represents the perivascular route of the CSF at the level of the large cerebral blood vessels at the base of the skull up to the olfactory bulb. Extraction of the lateral ventricles volume was performed manually using the Medical Image Processing, Analysis, and Visualization (MIPAV) software. See supporting information for details on gadolinium enhancement quantification.

^1^H‐MRS spectra were analyzed using LCModel version 6.3 1‐R.^25^ The LCModel results were reported as concentrations of metabolites. Details regarding MRS spectra quantification and the scaling of metabolite concentration in mmol/L have been reported in the supporting information.

### 2.8 Proteomic analysis

The experimental setting for sample preparation for proteomic analysis has been reported in detail in the supporting information. Briefly, CSF samples (20 µL), collected from each animal, were prepared for proteomic analysis using the ENRICH‐iST kit (PreOmics) following the manufacturer's instruction. Peptide concentration was used for data normalization. Raw mass spectra were then processed using MaxQuant software (v. 1.6.1.0)^26^ for label‐free protein quantification based on the precursor intensity.

The full dataset of identified and quantified proteins was further analyzed using a custom script developed in the R programming language. The mass spectrometry proteomics data have been deposited to the ProteomeXchange Consortium via the PRIDE.^27^

Differential analysis was performed for the comparison of DSS‐treated versus control mice samples. Estimated *P* values were corrected for multiple hypothesis testing (false discovery rate). Proteins with an absolute log fold change (logFC) > 1 and an adjusted *P* value < 0.05 were considered significantly differentially expressed.

Enrichment analysis on the subset of significant proteins was performed using the enrichR package;^28^ GO:BP (GO_Biological_Process_2023), GO:CC (GO_Cellular_Component_2023), KEGG (KEGG_2019_Mouse), and REACTOME (Reactome_2022) were used as annotations (databases already implemented in enrichR).

### 2.9 Western blotting for amyloid oligomers

Proteins were extracted from samples by Halt Protease and Phosphatase Inhibitor Single‐Use Cocktail, EDTA‐Free (Thermo Fisher Scientific, USA), followed by sonication, and lysed on ice for an additional 30 minutes. The cortical homogenates were centrifuged at 10000 × g for 10 minutes at 4°C, and the resulting supernatants were transferred to clean tubes. Protein concentrations were detected using the Pierce BCA Protein Assay Kit (Thermo Fisher Scientific) and quantified with a Multiskan FC Microplate Spectrophotometer (Thermo Fisher Scientific) at a wavelength of 570 nm. For western blot analysis, 70 µg of samples were loaded onto 4% to 15% Mini‐PROTEAN TGX Stain‐Free Protein Gels (Bio‐Rad Laboratories Inc., Italy) for electrophoresis. Thereafter, the separated proteins were transferred onto the nitrocellulose membranes (Bio‐Rad Laboratories Inc.) using the Pierce Power Blotter (Thermo Fisher Scientific). Membranes were blocked with 5% skimmed milk prepared in phosphate buffered saline (PBS) containing 0.1% Tween 20 (PBST) for 1 hour at room temperature and then were incubated overnight at 4°C with the following primary antibodies: rabbit polyclonal anti‐amyloid oligomers (ab126892, 1:500, Abcam) and mouse monoclonal anti‐β‐actin (ab8226, 1:1000, Abcam). Then, membranes were incubated for 1 hour at room temperature with the appropriate horseradish peroxidase (HRP)‐conjugated secondary antibodies: goat anti‐rabbit immunoglobulin G (IgG; ab6721, 1:3000, Abcam) or goat anti‐mouse IgG (ab205719, 1:5000, Abcam, UK). Between applications of different primary antibodies, membranes were stripped by Restore Western Blot Stripping Buffer (Thermo Fisher Scientific) for 10 minutes at room temperature. The protein expression was activated using Clarity Western ECL Substrate and analyzed by ChemiDoc XRS+ and Bio‐Rad Image Lab software version 5.2.1 (Bio‐Rad Laboratories Inc.). Optical densities of the bands were measured using ImageJ software (version 1.52, National Institutes of Health) and then normalized against β‐actin bands. Full gel is available as supplementary information (Figure 4E_supplementary data WB). Antibodies used are detailed as supplementary data (extended data table antibody). The rabbit polyclonal anti‐amyloid oligomers antibody, being conformation‐specific to A11, is effective in detecting amyloid oligomers. Therefore, according to literature studies,^29^ we quantified the highest band corresponding to oligomers (≈ 50 kDa), which could definitely not be monomers, fibrillar forms, cleavage products, or full‐length amyloid precursor protein.

### 2.10 Immunohistochemistry for phosphorylated tau

Mouse brains were washed in 0.9% NaCl and post‐fixed by immersion for 7 hours in 4% paraformaldehyde in 0.1 M phosphate buffer (pH 7.4), dehydrated through a graded ethanol series, and finally embedded in Paraplast X‐TRA 5 µm‐thick sections were cut in the transverse plane and collected on polylysine‐coated slides. After hematoxylin–eosin staining, brain slides of interest were selected. Slices were hydrated by performing a descending alcoholic scale. The tissue sections were dipped in a 10% H_2_O_2_ solution for 12 minutes to block endogenous peroxidase, and then 3 x 5 minute washes with PBS were performed. Slices were blocked by using the VECTASTAIN Elite ABC‐HRP Kit, Peroxidase (Rabbit IgG) and the VECTASTAIN Elite ABC‐HRP Kit, Peroxidase (Mouse IgG; Vector Laboratories). The slides were incubated overnight at 4°C with the primary antibody, rabbit anti‐p‐tau, diluted 1:100 in PBS. The next day, the tissue slices were washed with PBS 3 x 5 minutes, and then they were incubated with the anti‐rabbit secondary biotinylated antibodies (VECTASTAIN Elite ABC‐HRP Kit, Peroxidase [Rabbit IgG] and the VECTASTAIN Elite ABC‐HRP Kit, Peroxidase [Mouse IgG]; Vector Laboratories) for 30 minutes. After an additional three washes with PBS, slices were incubated with the streptavidin and biotin solution (Vector Laboratories) for 1 hour. After activation with H_2_O_2_, 3,3′‐diaminobenzidine ([DAB]; Sigma‐Aldrich) was added to the slices for 10 minutes to obtain the colorimetric reaction with the streptavidin–biotin–secondary antibody complex. The tissue sections were stained with hematoxylin–eosin and dehydrated through an ascending alcoholic scale. The slides were mounted using the Eukitt mounting medium (Sigma‐Aldrich), and then they were dried overnight before proceeding to microscope analysis. Microscopic image analysis was carried out by using Fiji ImageJ according to the Crowe and Yue protocol.^30^ Antibodies used are detailed as supplementary data (extended data table antibody).

### 2.11 Superfusion experiments

Purified isolated nerve endings (synaptosomes) and astrocytic specializations (gliosomes) were isolated from frozen prefrontal cortices and hippocampi as previously described.^31^ Briefly, tissues were homogenized in 0.32 M sucrose, buffered to pH 7.4 with tris‐(hydroxymethyl)‐amino methane (Tris, final concentration 0.01 M) using a glass/Teflon tissue grinder (clearance 0.25 mm). The homogenates were centrifuged at 1000 × g for 5 minutes to remove nuclei and cellular debris, and the supernatants were stratified on a discontinuous Percoll gradient (2%, 6%, 10%, and 20% v/v in Tris‐buffered sucrose) and centrifuged at 33500 × g for 6 minutes. The layers between 10% and 20% Percoll (synaptosomal fraction) and between 6% and 2% Percoll (gliosomal fraction) were collected and washed by centrifugation at 19000 × g for 15 minutes in a physiological medium having the following composition (mM): NaCl, 140; KCl, 3; MgSO_4_, 1.2; CaCl_2_, 1.2; NaH_2_PO_4_, 1.2; NaHCO_3_, 5; HEPES, 10; glucose, 10; pH 7.4.

Release experiments were carried out with an experimental approach, the “superfusion of a thin layer of synaptosomes.”^29^ Suspended synaptosomes and gliosomes were incubated for 15 minutes at 37°C in a rotating water bath in the presence of [^3^H]D‐aspartate (^3^[H]D‐ASP, f.c. 50 nM; NET581001MC, Perkin Elmer), an unmetabolizable analogue of glutamate, or [^3^H]D‐g‐aminobutyric acid (^3^[H]GABA, f.c. 30 nM). In the experiments of GABA release, 50 µM aminooxy acetic acid was added to the medium to prevent GABA metabolism. [^3^H]D‐ASP is routinely used as a marker of the endogenous amino acid in release studies.^32^ Identical aliquots of the synaptosomal or gliosomal suspensions were then stratified on microporous filters at the bottom of parallel chambers in a Superfusion System^32^ and kept at 37°C and superfused with the standard physiological solution at 0.5 mL/minute. After 39 minutes of superfusion to equilibrate the system, synaptosomes and gliosomes were transiently exposed (90 seconds) to a KCl‐enriched medium (15 mM KCl‐containing medium). Four 3 minute superfusate fractions were collected starting from t = 36 minutes of superfusion. The fractions and the superfused particles were then counted for radioactivity. The amount of radioactivity released into each fraction was expressed as a percentage of the total radioactivity. The evoked overflow was estimated by subtracting the neurotransmitter content in the first and the last basal fractions from that in the intermediate two fractions, with the exception of the results described for GABA and aspartate in hippocampus and cortex synaptosomes and gliosomes, for which data are expressed as induced overflow (%) over basal release.

### 2.12 Statistical analysis

All the data shown are expressed as mean ± standard error of the mean.

The relative densities of the acquired images from western blot immunodetection and immunohistochemistry were analyzed with ImageJ software. Statistical analyses were accomplished using Prism software (GraphPad, version 9.0). Statistical differences were determined by analysis of variance (ANOVA) and, when significant, by an appropriate post hoc test. In all reported statistical analyses, differences were labeled as non‐significant for *P* > 0.05, significant (*) for *P* < 0.05, very significant (**) for *P* < 0.01, extremely significant (***) for *P* < 0.001 and (****) for *P* < 0.0001.

Statistical evaluation (GraphPad Software Inc.) related to MRI SE (%) was performed by applying a two‐way ANOVA mixed‐effect model for repeated measures with Bonferroni multiple comparisons test.

Statistical analysis for western blot analysis of brain proteins and release experiments was carried out by a direct comparative Student *t* test.

Regarding behavioral tests, each measurement was taken from a distinct animal. Estimates of Cohen *d* effect sizes were calculated by using G power 3.1.9.7 software. Considering sample sizes, Cohen *d* effect size calculation results for the behavioral test were 1.54 (sample size group 1 = 10, sample size group 2 = 15), 1.56 (sample size group 1 = 10, sample size group 2 = 14), 1.59 (sample size group 1 = 10, sample size group 2 = 13), and 1.66 (sample size group 1 = 10, sample size group 2 = 11). Such values are in line with the literature related to preclinical experiments, in which a higher standard effect size *d* is commonly reported.^33^ In particular, because laboratory animals are intrinsically much more uniform than humans, and also taking into account ethical issues, effect size *d* can be estimated by considering “extra‐large” (1.1), “gigantic” (1.5), or “awesome” (2.0) values.^33^ Data were tested for normality by performing Shapiro–Wilk or Kolmogorov–Smirnov normality tests, and, subsequently, two‐tailed unpaired Student *t* test, with Welch correction when needed, or Mann–Whitney test, were used to analyze them.

## 3 RESULTS

### 3.1 Characterization of the acute DSS‐induced colitis model

Mice receiving 2.5% (w/v) DSS (Figure 1A) showed significantly higher DAI compared to control, along with significantly reduced colon length and body weight (Figure 1B). DSS‐treated mice showed altered colon architecture (Figure 1C) and intestinal barrier integrity (Figure 1D), accompanied by colonic inflammation (Figure 1E) and increased aquaporin‐4 (*Aqp4*) mRNA, consistent with previous evidence (Figure 1F).^34^, ^35^ Markers of systemic inflammation were detected in serum (Figure S2A in supporting information), indicating that colitis drives a global inflammatory response that finally results in neuroinflammation (Figure S2B–D).

A significant increase in visceral sensitivity in response to CRD was evident after the 72 hour washout from the DSS challenge (Figures 1G and S3 in supporting information), together with a significant decrease in the somatic pain threshold measured on the paw by thermal and mechanical stimuli (Figure 1H).

We further analyzed animal behavior. After excluding locomotor activity impairments (Figure 1Ia), we found that DSS‐treated mice were characterized by anxiety‐like behavior (Figure 1I), whereas depressive‐like behavior and cognitive impairment were not retrieved (Figure S4 in supporting information). However, an important limitation of this study is that we cannot exclude a potential contribution of visceral pain to the observed anxiety‐like behaviors. Therefore, future studies are warranted to disentangle the contributions of pain and inflammation to the anxiety‐like phenotype observed in DSS‐treated mice.

### 3.2 Misalignment of circadian clockwork from periphery to center

**FIGURE 2:**
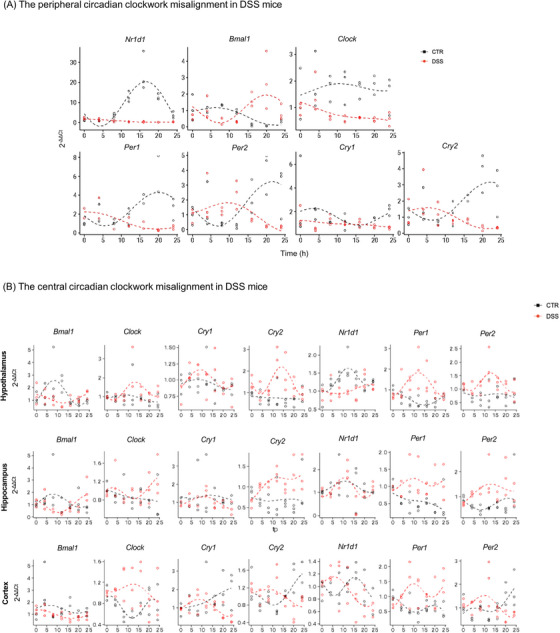
Misalignment of circadian clockwork from periphery to center. A, Scatter plot of the expression (2^‐ΔΔCt) of different clock genes across different time points (hours) in the colon dataset. The color indicates the assignment of the data point to a specific condition. The dashed lines represent the modeled gene expression in each condition across time. The modeling has been performed using the MetaCycle package (*n* = 3 for each time point). B, Scatter plot of the expression (2^‐ΔΔCt) of different clock genes across different time points (hours) in the hippocampus, hypothalamus, and cortex datasets. The color indicates the assignment of the data point to a specific condition. The dashed lines represent the modeled gene expression in each condition across time. The modeling has been performed using the MetaCycle package. *n* = 3 for each time point. CTR, control; DSS, dextran sulfate sodium

Several studies related the loss of different circadian clock components to IBD pathogenesis, suggesting that circadian clock genes are important regulators of colitis.^14^, ^36^ We then investigated expression of core circadian clock genes nuclear receptor subfamily 1 group D member 1 (*Nr1d1*), basic helix‐loop‐helix ARNT‐like protein 1 (*Bmal1*), circadian locomotor output cycles kaput (*Clock*), period circadian protein homolog 1 (*Per1*), period circadian clock 2 (*Per2*), cryptochrome circadian regulator 1 (*Cry1*), and cryptochrome circadian regulator 2 (*Cry2*) in colon tissues using qPCR. These specific clock genes are essential for the proper functioning of the circadian clock. Indeed, the circadian clock runs on an intricate system of interconnected feedback loops, primarily driven by the partnership of the two positive regulators *BMAL1* and *CLOCK*, working together to activate the production of the negative regulators *PER* and *CRY*. Beyond this core loop, other vital regulators, like *NR1D1*, contribute to the overall oscillatory mechanism. The transcription of these genes oscillated in the colon tissue, indicating clock rhythmicity at the molecular level. After the DSS insult, the clock genes are desynchronized compared to the control group for all the ZT times (Figure 2A). These results showed that the DSS challenge strongly disrupted the rhythm of clock genes in the colon. Moreover, mRNA levels of all clock genes, except for *Bmal1*, were significantly lower in the inflamed colon compared to controls (Figure S5A in supporting information).

As observed in the periphery, a misalignment in the circadian intracellular machinery has been found in brain areas, such as the hypothalamus, the central regulator of the circadian rhythm, the hippocampus, and the cortex, important areas for the analyzed behaviors, from DSS‐mice compared to controls, even if with a different pattern of clock gene expression compared to the colon (Figures 2B and S5B). These results suggest that an imbalance in circadian regulatory mechanisms can be transferred from the periphery to the CNS, thereby affecting several functions regulated by circadian rhythms. Among these, the glymphatic system, regulating brain fluid dynamics, has been shown to be under circadian control.^12^

### 3.3 Alteration in brain fluid dynamics in DSS‐treated mice by in vivo imaging

**FIGURE 3:**
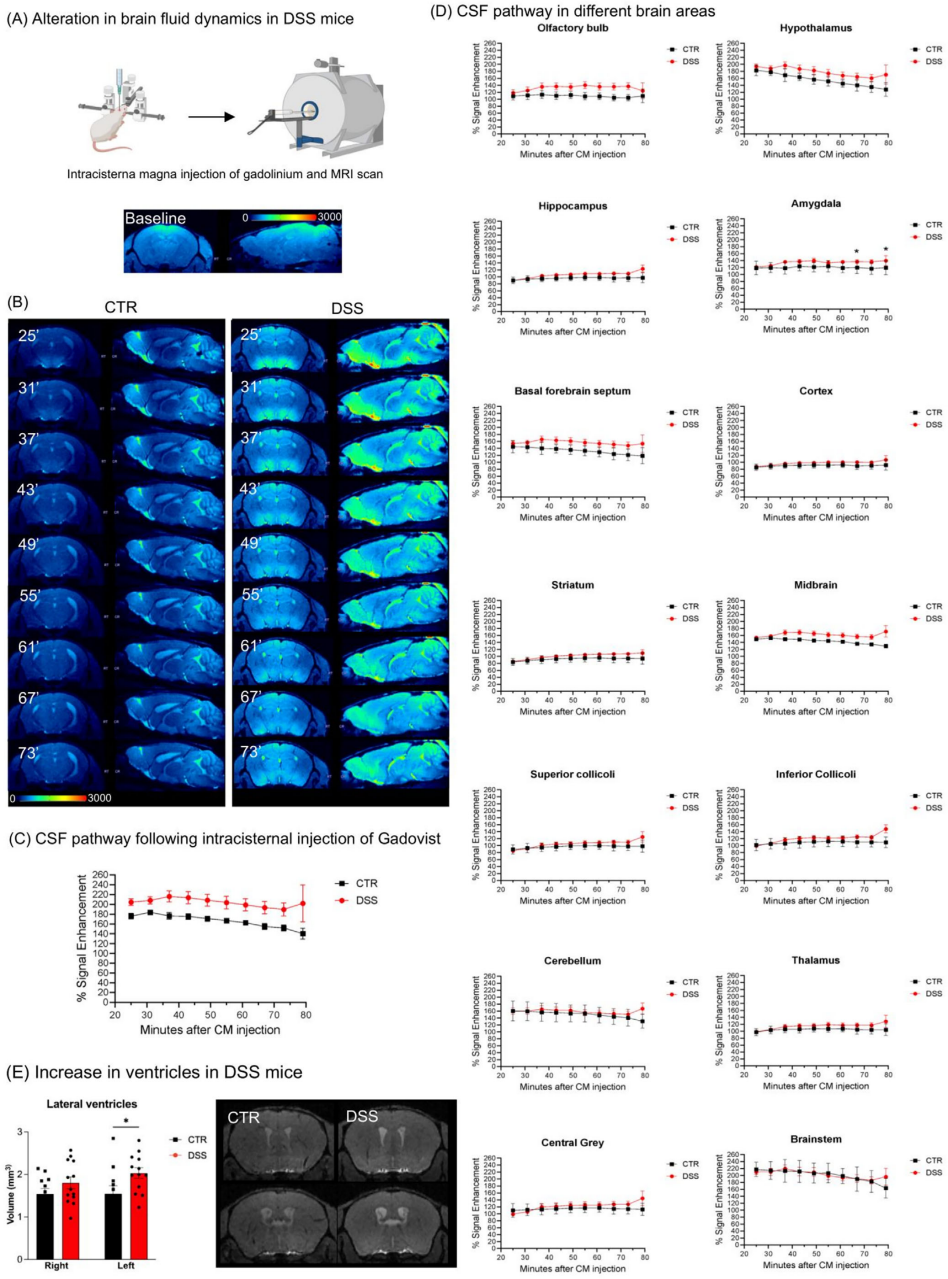
Alteration in brain fluid dynamics in acute DSS‐induced colitis mice. A, Cartoon of the intracisterna magna injection of gadolinium and MRI scan. B, Representative longitudinal 3D T1‐weighted high‐resolution images of gadolinium‐enhanced MRI of the brain before (baseline upper panel) and after intracisternal injection of Gadovist in a CTR (lower left panel) and a DSS‐treated mouse (lower right panel) under ketamine/xylazine general anesthesia. C, The percentage SE (%)—with respect to the baseline as a function of time after the intracisternal (CM) injection of Gadovist in CTR and DSS‐treated animals. A significant general effect on condition was found (*P* < 0.0001). Group data were gathered every 6 minutes. The manually defined region of interest was the perivascular pathway of the CSF along the brain vessels. Sample size for each time point includes CTR *n* = 9 for 25′, *n* = 11 for 31′, 37′, 43′, 49′, 55′, 61′; *n* = 11 for 67′, 73′; *n* = 5 for 79′; DSS *n* = 12 for 25′, 31′; *n* = 13 for 37′, 43′, 49′, 55′, 61′, 67′; *n* = 12 for 73′; *n* = 4 for 79′. Data are expressed as mean ± SEM and analyzed using a two‐way ANOVA with Bonferroni post hoc test. D, The percentage SE (%)—with respect to the baseline as a function of time after the intracisternal (CM) injection of Gadovist in CTR and DSS‐treated animals in brain regions extracted from a predefined mouse atlas. Group data were gathered every 6 minutes. The analyzed regions were: olfactory bulb, hypothalamus, hippocampus, amygdala, basal forebrain septum, cortex, striatum, midbrain, superior and inferior colliculi, brainstem, cerebellum, thalamus, and central gray (CTR *n* = 9 for 25′, *n* = 11 for 31′, 37′, 43′, 49′, 55′, 61′, 67′, 73′; *n* = 5 for 79′; DSS *n* = 12 for 25′, 31′; *n* = 13 for 37′, 43′, 49′, 55′, 61′, 67′; *n* = 12 for 73′; *n* = 4 for 79′). Significant general effects on condition were found for the olfactory bulb (*P* = 0.048), striatum (*P* = 0.020), hippocampus (*P* = 0.023), basal forebrain septum (*P* = 0.027), hypothalamus (*P* = 0.045), amygdala (*P* = 0.016), and midbrain (*P* = 0.011). Data are expressed as mean ± SEM and analyzed using a two‐way ANOVA (condition and time post intracisternal Gadovist injection were considered variables, and a mixed effect model was applied for missing values) with Bonferroni post hoc test (*P < 0.05, **P < 0.01, ***P < 0.001 vs. respective control). E, Lateral ventricle volume from CTR and DSS‐treated animals extracted from the last time point after intracisternal (CM) injection of Gadovist (73′ post injection) by manually drawing a VOI of the left and the right lateral ventricles; CTR *n* = 11, DSS *n* = 13; Data are expressed in mm^3 as mean ± SEM and analyzed using a two‐way ANOVA (condition and hemisphere were considered variables) with Bonferroni post hoc test (*P < 0.05, **P < 0.01, ***P < 0.001 vs. respective control). ANOVA, analysis of variance; CSF, cerebrospinal fluid; CTR, control; DSS, dextran sulfate sodium; MRI, magnetic resonance imaging; SE, signal enhancement; SEM, standard error of the mean; VOI, volume of interest

To assess brain fluid distribution dynamics, we performed multiple 3D T1‐weighted MRI acquisitions until 1.5 hours post intracisternal injection of the gadolinium‐based contrast agent Gadovist (Figure 3A), serving as a tracer of CSF transport and tissue uptake.^37^ After injection into the cisterna magna, the tracer spread throughout the entire brain, inducing an alteration of the intrinsic baseline contrast. Clear perfusion differences between the controls and DSS‐treated mice were observed (Figure 3B). The presence of the tracer was significantly higher along the CSF perivascular pathway after DSS exposure (Figure 3C). SE (%) was significantly increased as a general effect on condition in the olfactory bulb, striatum, hippocampus, hypothalamus, amygdala, basal forebrain septum, and midbrain of DSS‐treated mice compared to controls (Figure 3D). A *post hoc* test indicated in the DSS model a SE (%) significant increase in the amygdala (*P* = 0.046 at 61′ and 73′ post‐injection). In the other brain regions (cortex, thalamus, cerebellum, midbrain, superior colliculi), a trend, even if not reaching statistical significance, of SE (%) increase was always observed. These results demonstrated how the contrast agent concentration was increased and the clearance delayed in DSS‐exposed mice, thus suggesting impaired brain fluid circulation. Furthermore, a statistically significant enlargement of lateral ventricle volume was observed in DSS‐treated mice compared to controls (Figure 3E), an index of an altered brain structure morphology, possibly involving altered compliance to pressure and fluid volume changes. However, the analysis of the nuclei in the periventricular area, as well as in other brain regions, did not show alteration in cellular parameters, such as chromatin integrity and cellular morphology, thus indicating no signs of neurodegeneration associated with the ventricle enlargement (data not shown). Altogether, in animal models exposed to an acute intestinal inflammatory insult, an alteration in brain fluid dynamics circulation occurs, possibly impairing cerebral drainage systems, like the perivascular/glymphatic pathway.

### 3.4 Alteration in brain CSF protein content and aberrant deposition of waste products in mice with acute DSS‐induced colitis

**FIGURE 4:**
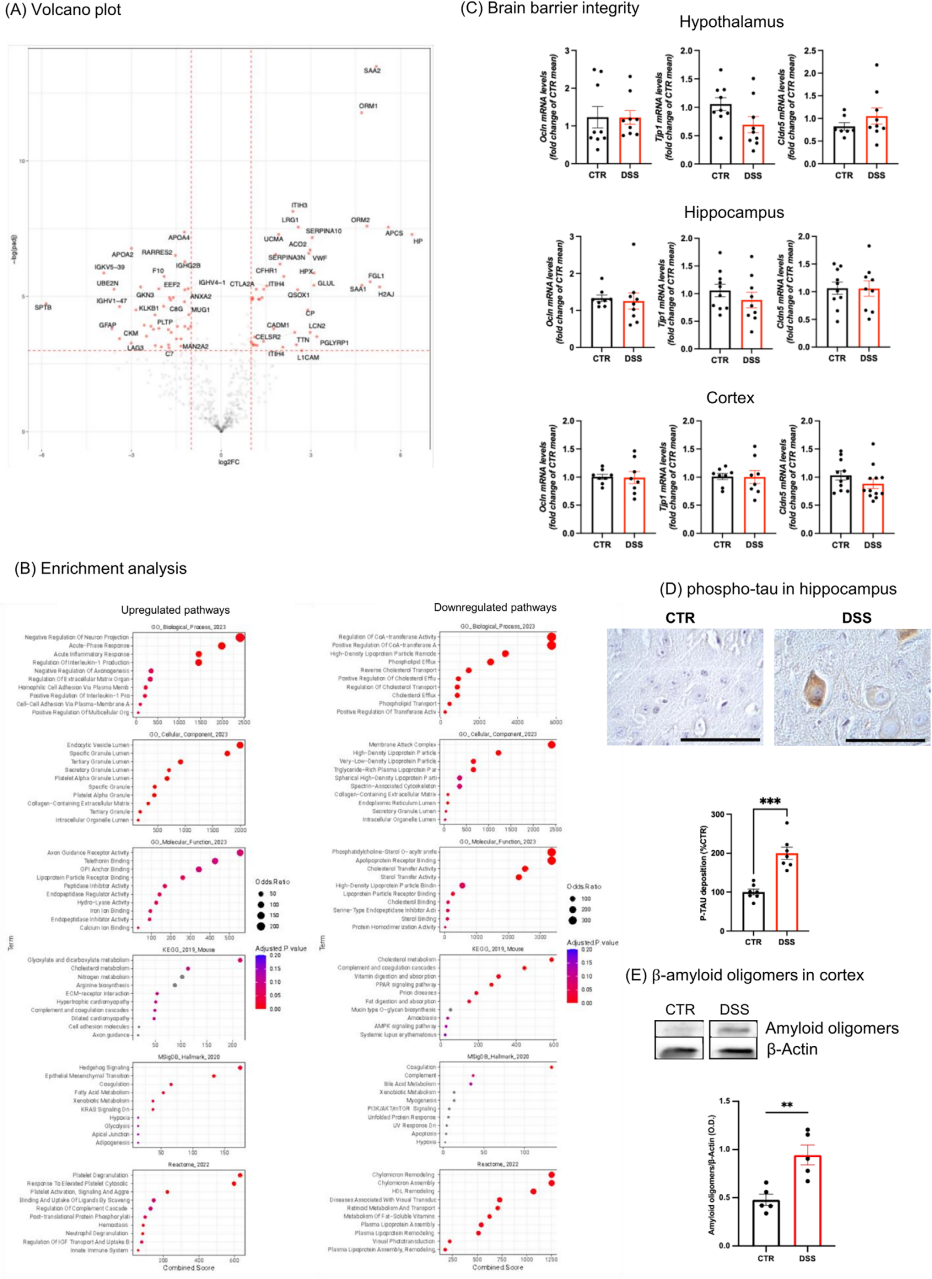
Alteration in brain CSF protein content and aberrant waste product deposition in acute DSS‐induced colitis mice. A, Volcano plot of the proteomic data analysis on CSF from control and DSS mouse model (CTR = 8; DSS = 8). The red dots identify the significant differentially regulated proteins in the dataset (absolute log2FC > 1 and adjusted *P* value < 0.05). A positive FC indicates more expression in the DSS condition. B, Enrichment analysis results run over the significant proteins (divided by upregulated or downregulated). We reported the top 10 terms per annotation ranked by combined score. On the *X* axis, we reported the combined score (a metric derived from enrichR); on the *Y* axis, the name of the term. We divided the plot per annotation. Here, we have reported the results from Gene Ontology Biological Processes, Gene Ontology Cellular Component, KEGG and REACTOME, Gene Ontology Molecular Function, MSigDB hallmark, and Wiki Pathway. C, mRNA expression of the blood–brain barrier junctions *Ocln*, *Tjp1*, and *Cldn5* was measured in the hypothalamus, hippocampus, and cortex (hypothalamus *Ocln* and *Tjp1* *n* = 9/group, *Cldn5* CTR *n* = 7 and DSS *n* = 9; hippocampus *Ocln* CTR *n* = 8 and DSS *n* = 9, *Tjp1*, and *Cldn5* CTR *n* = 10 and DSS *n* = 9; cortex *Ocln* and *Tjp1* *n* = 8/each group and *Cldn5* CTR *n* = 11 and DSS *n* = 12) by rt‐PCR. Data are reported as mean ± SEM and analyzed using an unpaired two‐tailed *t* test. D, Phosphorylated tau protein density in hippocampus from control (CTR, black bar) and DSS (red bar) mice. Representative images of phosphorylated tau for the CTR and DSS conditions are reported; magnification 100×; scale bar 100 µm. Immunohistochemistry was performed on brains of both CTR (*n* = 7) and DSS‐treated mice (*n* = 7) using anti‐phosphorylated tau. Data are expressed as the mean percentage of phosphorylated tau deposition in brains. Two‐tailed unpaired Student *t* test. ***P < 0.001 DSS versus CTR. E, Amyloid oligomer protein density in cortical homogenates from control (CTR, black bar) and DSS (red bar) mice. Amyloid oligomers protein density was expressed as the protein/β‐actin ratio and changes in DSS mice were expressed as the O.D. of the protein/β‐actin ratio in CTR mice. The panel shows representative images in homogenates from the cortex of CTR mice (*n* = 5) and DSS mice (*n* = 5). Data are expressed as mean ± SEM. Two‐tailed unpaired Student *t* test, *t* = 3.922, df = 8, C.I. = −0.7382 to −0.1915, η^2^ = 0.6578, *P* = 0.0044, **P < 0.01 DSS versus CTR. CSF, cerebrospinal fluid; CTR, control; DSS, dextran sulfate sodium; FC, fold change; O.D., optical density; rt‐PCR, reverse transcription polymerase chain reaction; SEM, standard error of the mean

Based on data of alteration in brain fluid dynamics circulation, we investigated whether differences in the content of circulating CSF were observed in acute DSS‐induced colitis mice compared to controls. Quantitative proteomics was performed to explore the protein composition of CSF from single‐animal samples from eight DSS‐treated and eight control animals. We identified 608 proteins by nano‐liquid chromatography tandem mass spectrometry proteomic analysis, and 110 proteins were found significantly dysregulated out of 502 quantified proteins between the two groups. Differentially expressed proteins from DSS‐treated mice versus controls were shown in a volcano plot (Figure 4A) and used to run different enrichment analyses (Figure 4B). Enrichment analysis revealed that the CSF from DSS‐treated mice was enriched in proteins associated with inflammatory pathways, immune response, complement, cell–cell adhesion molecules, neuronal pathways, and lipid metabolism (Figure 4B).

Despite the lower cerebral drainage functionality and changes in brain structure, DSS‐treated mice did not show a statistically significant difference in gene expression of occludin *(Ocln*), tight junction protein ZO‐1 (*Tjp1*), and Claudin‐5 (*Cldn5*), involved in brain barrier integrity and analyzed in the hypothalamus, hippocampus, and cortex (Figure 4C). However, as a consequence of the circulatory stasis of brain fluids, deposition of waste products has been observed mainly in the cortex and hippocampus of DSS‐treated mice. Indeed, in the DSS‐driven acute colitis model, the peripheral insult significantly promoted an increase in phosphorylated tau deposition in the hippocampus (Figure 4D), as well as an increased deposition in amyloid oligomers in the cortex (Figure 4E).

### 3.5 Glial immunoreactivity in acute DSS‐induced colitis mouse brains

**FIGURE 5:**
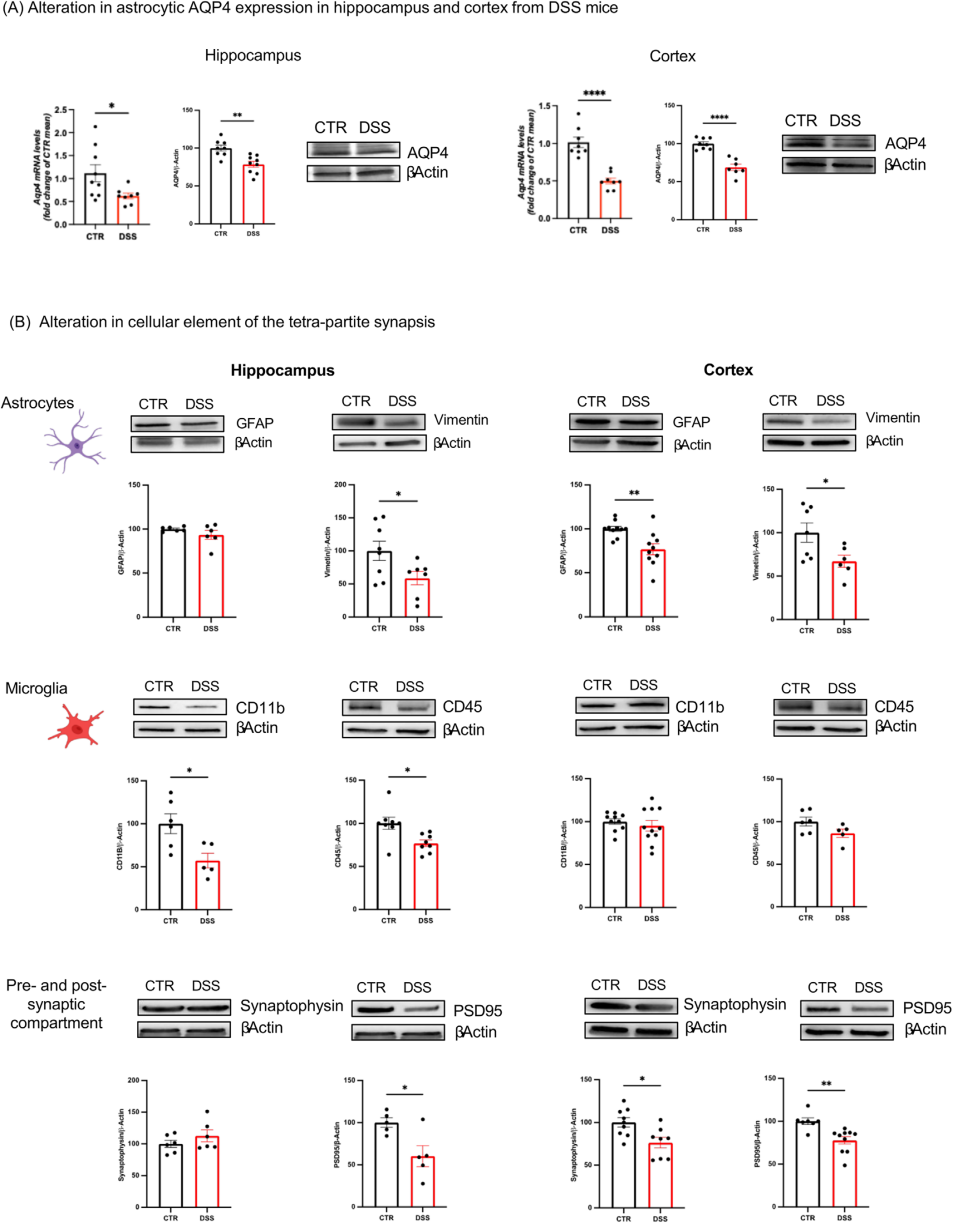
Glial immunoreactivity in acute DSS‐induced colitis mouse brains. A, AQP4 mRNA and protein expression in hippocampal (left) and cortical (right) samples from control (CTR, black bar) and DSS (red bar) mice is shown. AQP4 protein density was expressed as protein/β‐actin ratio and changes in DSS mice were expressed as a percentage of the protein/β‐actin ratio of CTR mice. β‐Actin was used as an internal standard. PCR data are reported as mean ± standard error of the mean and analyzed using an unpaired two‐tailed *t* test (*P < 0.05 and ****P*p* < 0.0001 vs. respective controls). The panel shows representative images of AQP4 immunopositivity of homogenates from CTR mice (hippocampus, *n* = 8; cortex, *n* = 8) and DSS mice (hippocampus, *n* = 9; cortex, *n* = 7). Two‐tailed unpaired Student *t* test, *P < 0.05, **P < 0.01, ***P < 0.001, ****P < 0.0001, versus respective control. B, Protein densities of the principal components of the tetra‐partite synapses in DSS mice compared to CTR. Astrocytes: GFAP and vimentin protein immunoblotting (up) and density (down) in hippocampal (left) and cortical (right) homogenates from CTR mice (black bar, GFAP: hippocampus, *n* = 6; cortex, *n* = 10; vimentin: hippocampus, *n* = 8; cortex, *n* = 7) and DSS mice (red bar, GFAP: hippocampus, *n* = 6; cortex, *n* = 10; vimentin: hippocampus, *n* = 7; cortex, *n* = 6). Microglia: CD11b and CD45 protein immunoblotting (up) and density (down) in hippocampal (left) and cortical (right) homogenates from CTR mice (black bar, CD11b: hippocampus, *n* = 6; cortex, *n* = 10; CD45: hippocampus, *n* = 8; cortex, *n* = 6) and DSS mice (red bar, CD11b: hippocampus, *n* = 5; cortex, *n* = 11; CD45: hippocampus, *n* = 8; cortex, *n* = 5). Pre‐ and post‐synaptic compartments: Synaptophysin and PSD95 protein immunoblotting (up) and density (down) in hippocampal (left) and cortical (right) homogenates from CTR mice (black bar, Synaptophysin: hippocampus, *n* = 6; cortex, *n* = 9; PSD95: hippocampus, *n* = 5; cortex, *n* = 7) and DSS mice (red bar, Synaptophysin: hippocampus, *n* = 6; cortex, *n* = 8; PSD95: hippocampus, *n* = 5; cortex, *n* = 10). The density of each protein was expressed as a protein/β‐actin ratio; changes in DSS mice were expressed as a percentage of the protein/β‐actin ratio in CTR mice. β‐Actin was used as an internal standard. The panel shows representative images for each investigation. Two‐tailed unpaired Student *t* test, *P < 0.05, **P < 0.01, versus respective control. Full gels are available as supporting information (Figure 5_supplementary data WB). AQP4, aquaporin‐4; DSS, dextran sulfate sodium; GFAP, glial fibrillary acidic protein; PCR, polymerase chain reaction; PSD95, postsynaptic density protein 95

To acquire more insight into brain fluid circulation imbalance, we analyzed the expression of AQP4 as a key player of the glymphatic system^38^ and main marker of astrocytic end‐feet. In DSS‐insulted mice, we found decreased AQP4 expression in both the hippocampus and cortex, compared to control animals (Figure 5A).

It is noteworthy that the significant reduction of AQP4 in the cortex and hippocampus might suggest reduced connections with either capillaries or neurons, potentially leading to decreased energy supply. This, in turn, could affect energy‐dependent neuronal processes, including neurotransmitter exocytosis. Furthermore, astrocytic expression has been completed by glial fibrillary acidic protein (GFAP) and vimentin analysis. GFAP, used to detect astrocytes in the white matter,^39^ has been found significantly reduced at the cortical but not at the hippocampal level (Figure 5B) in DSS‐treated mice compared to controls. On the contrary, vimentin, a marker of astrocytes in the gray matter,^40^ showed significantly decreased expression levels both in the hippocampus and cortex (Figure 5B) of DSS mice. These results suggest a rearrangement of the connections and distribution of astrocytes in the gray area to support the concept of the tetra‐partite synapse as a specific target of “gut‐to‐brain” insults, particularly in terms of altered astrocyte–neuron communication. Moreover, imbalance in microglia and pre‐ and post‐synaptic components has also been observed in DSS‐treated mice. On one side, cluster of differentiation 11B (CD11b) and cluster of differentiation 45 (CD45) expression have been found significantly decreased in the hippocampus, but not at the cortical level (Figure 5B). Although these proteins can also be expressed by macrophages, they both are widely used to monitor microglia activation in inflammatory conditions.^41^ On the other side, when analyzed for synaptophysin and postsynaptic density protein 95 (PSD95), synaptophysin showed reduced expression in the cortex but not in the hippocampus, whereas PSD95 expression was found to be decreased in two brain areas of DSS‐treated mice compared to controls (Figure 5B). These data are consistent with the potential impact of DSS either on the functionality (i.e., the efficiency of presynaptic exocytosis) or on the structural connectivity (i.e., the bridging efficiency of PSD95 to stabilize the synaptic cleft) of the tetra‐partite synapse, as detailed in the following section.

### 3.6 Altered synaptic transmission in acute DSS‐induced colitis mouse brains

**FIGURE 6:**
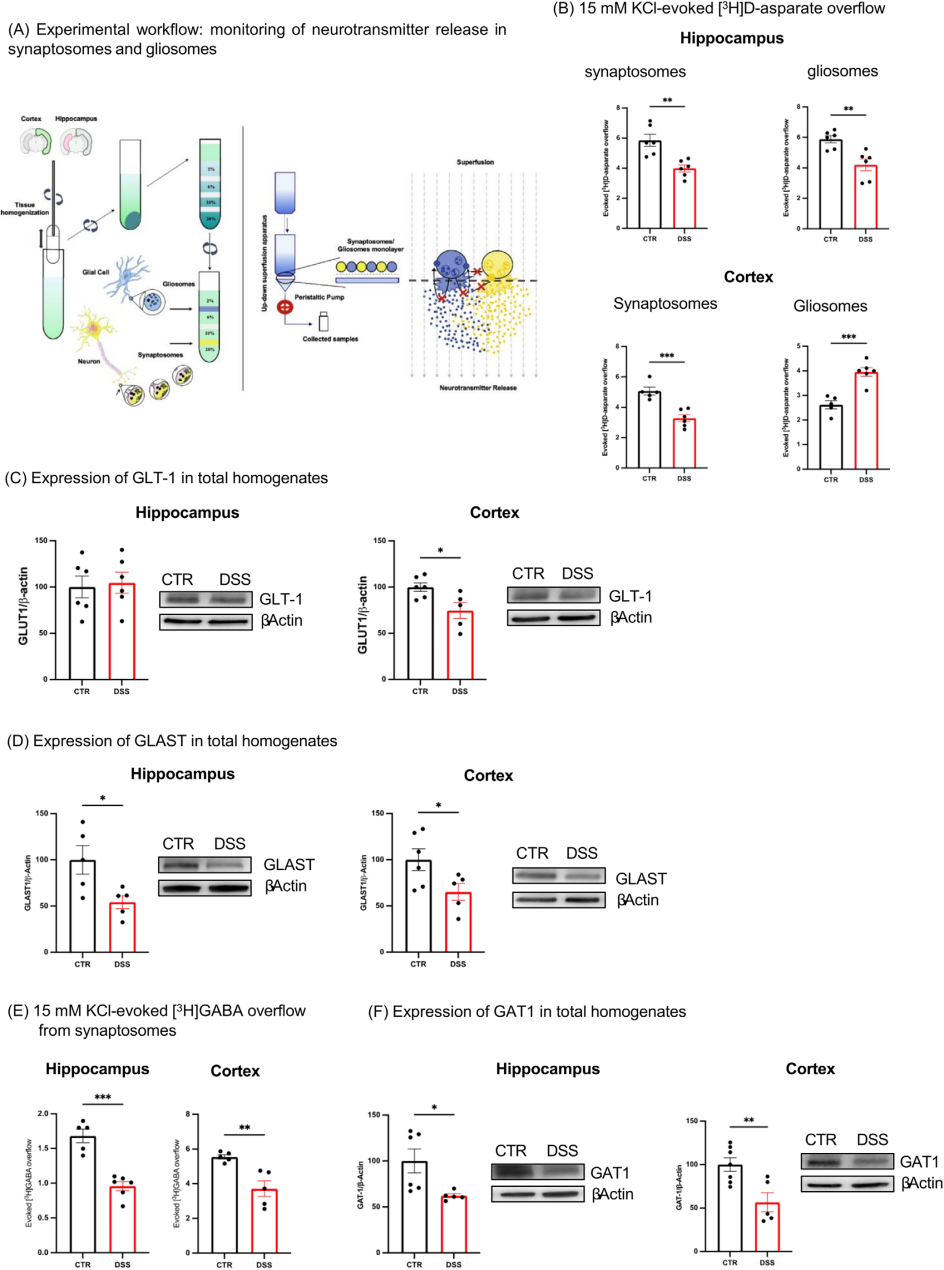
Altered synaptic transmission in acute DSS‐induced colitis mouse brains. A, Cartoon of the (i) technique of isolation of purified nerve endings (synaptosomes) and astrocytic specializations (gliosomes) from hippocampal and cortical tissues and their purification on Percoll gradient and (ii) of the “up–down superfusion technique” to monitor neurotransmitter release. B, Release of preloaded [^3^H]D‐aspartate evoked by 15 mM KCl from hippocampal synaptosomes (up, left, *n* = 6), cortical synaptosomes (down, left, *n* = 5), hippocampal gliosomes (up, right, *n* = 6), and cortical gliosomes (down, right, *n* = 5) of control (CTR, black bar) and DSS mice (red bar). The tritium overflow is calculated as [^3^H]D‐ASP overflow over basal release, and it is expressed as a percentage of total tritium content in both particles. Data are expressed as mean ± SEM of (*n*) experiments run in triplicate on different days. Two‐tailed unpaired Student *t* test, *P < 0.05, **P < 0.01, ***P < 0.001 versus respective control. C, GLT‐1 protein density in hippocampal (left) and cortical (right) homogenates from control (CTR, black bar) and DSS (red bar) mice. GLT‐1 protein density was expressed as a protein/β‐actin ratio, and changes in DSS mice were expressed as a percentage of the protein/β‐actin ratio in CTR mice. β‐Actin was used as an internal standard. The panel shows representative images of GLT‐1 immunopositivity of homogenates from CTR mice (hippocampus, *n* = 6; cortex, *n* = 6) and DSS mice (hippocampus, *n* = 6; cortex, *n* = 5). Two‐tailed unpaired Student *t* test, *P < 0.05, versus respective control. D, GLAST protein density in hippocampal (left) and cortical (right) homogenates from control (CTR, black bar) and DSS (red bar) mice. GLAST protein density was expressed as a protein/β‐actin ratio, and changes in DSS mice were expressed as a percentage of the protein/β‐actin ratio in CTR mice. β‐Actin was used as an internal standard. The panel shows representative images of GLAST immunopositivity of homogenates from CTR mice (hippocampus, *n* = 5; cortex, *n* = 6) and DSS mice (hippocampus, *n* = 5; cortex, *n* = 5). Two‐tailed unpaired Student *t* test, *P < 0.05, versus respective control. E, Release of preloaded [^3^H]‐GABA evoked by 15 mM KCl from hippocampal synaptosomes (left, *n* = 5) and cortical synaptosomes (right, *n* = 5) of control (CTR, black bar) and DSS mice (red bar). The tritium overflow is calculated as [^3^H]‐GABA overflow over basal release, and it is expressed as a percentage of total tritium content. Data are expressed as mean ± SEM of (*n*) experiments run in triplicate in different days. Two‐tailed unpaired Student *t* test, **P < 0.01, ***P < 0.001 versus respective control. F, GAT1 protein density in hippocampal (left) and cortical (right) homogenates from control (CTR, black bar) and DSS (red bar) mice. GAT1 protein density was expressed as a protein/β‐actin ratio, and changes in DSS mice were expressed as percentage of the protein/β‐actin ratio in CTR mice. β‐Actin was used as an internal standard. The panel shows representative images of GAT1 immunopositivity of homogenates from CTR mice (hippocampus, *n* = 6; cortex, *n* = 7) and DSS mice (hippocampus, *n* = 5; cortex, *n* = 5). Two‐tailed unpaired Student *t* test, *P < 0.05, **P < 0.01 versus respective control. Western blot analyses were performed on distinct samples on different days (number of samples = *n* as indicated). Full gels are available as supporting information (Figure 6_supplementary data WB). CTR, control; DSS, dextran sulfate sodium; GAT1, GABA transporter type 1; GLAST, glutamate aspartate transporter; GLT‐1, glutamate transporter 1; SEM, standard error of the mean

The impact of an acute peripheral insult on in vitro glutamate (Glu) and GABA KCl‐evoked exocytosis in synaptosomes and gliosomes of mouse hippocampus and cortex was investigated (Figure 6A). In the brains of mice with acute DSS‐induced colitis, Glu overflow triggered by 15 mM KCl was significantly reduced in both hippocampal synaptosomes and gliosomes, compared to controls. In the cortex, however, Glu exocytosis efficiency was reduced in synaptosomes but enhanced in gliosomes (Figure 6B). These data highlight that the response to a depolarizing stimulus, measured as efficiency of Glu exocytosis from nerve endings, is altered in an area‐dependent manner, with a predominance of astrocytic over nerve signals in the cortex of DSS mice. Furthermore, while the synaptic signal has been observed always reduced in both hippocampus and cortex, the astrocytic processes changed, becoming hyper‐ or hypo‐sensitive to a depolarizing stimulus depending on the area. Concomitant to adaptation in neurotransmitter exocytosis, we also observed changes in the expression of glutamate transporter 1 (GLT‐1) and glutamate aspartate transporter (GLAST). GLAST/GLT1 belong to the solute class 1 of transporters and have different area‐dependent cellular localization, the astrocytes being endowed with both transporters, while neuronal specializations, particularly the cortical ones, preferentially expressing the GLT‐1.^42^ We analyzed the density of both the transporters in the whole hippocampal and cortical homogenates. GLT‐1 has been found decreased at the cortical level (Figure 6C), whereas the expression of GLAST was reduced both in hippocampus and cortex (Figure 6D), thus suggesting the Glu reuptake mechanisms were also altered in DSS‐treated mice compared to control animals in a region‐ and subcellular‐dependent manner. In acute DSS induced‐colitis, the cortical reduction of GLT‐1 and GLAST transporters indicates that the Glu recovery mechanism is reduced, thus potentially leading to a prolonged excitatory state. When monitoring the release of preloaded [^3^H] GABA release evoked by KCl, GABA exocytosis from the synaptosomes and gliosomes was found reduced both in hippocampus and cortex of DSS‐treated mice (Figure 6E), thus altering the presynaptic mechanism of feedback control of excitatory glutamatergic signals. Moreover, also the expression of the GAT1 transporter was found decreased in the hippocampus and cortex of DSS mice (Figure 6F). Taking into account that [^3^H]GABA identifies the source of the newly taken‐up GABA accumulating in the readily releasable pool, the reduction of [^3^H]GABA exocytosis could be supported by decreased capacity of the nerve to pick up GABA and to replenish the vesicular reservoir that subserves rapid responses to stimuli.

### 3.7 Alterations in the levels of brain metabolites by in vivo MRS in DSS‐treated mice

**FIGURE 7:**
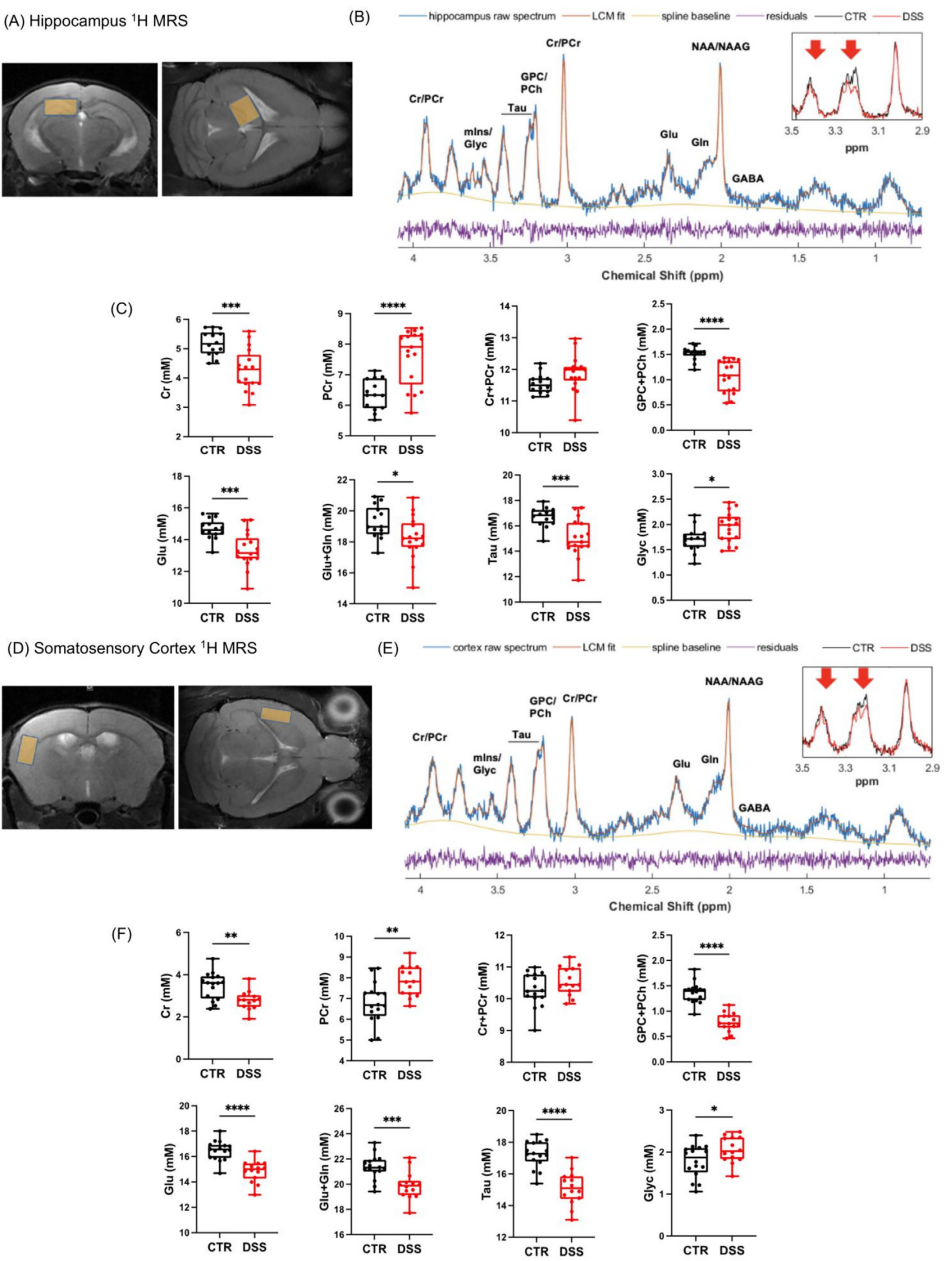
In vivo ^1^H MRS in CTR and DSS‐treated mice. A and D, Voxel location in the left hippocampus and in the left somatosensory cortex respectively, depicted over T2 RARE axial and coronal images of the brain. B and E, Representative spectra of average quality (light blue line) in the left hippocampus and left somatosensory cortex, respectively, fitted by LCModel (orange line). The spline baseline (yellow line) and the residuals (purple line) are also depicted. For each graph the inset is a zoom of two representative spectra, one CTR (black line) and one DSS (red line), in the region of major spectrum alterations (total choline and taurine) between 2.9 and 3.5 ppm. C and F, Boxplots of the concentrations of the metabolites of interest in mmol/L in CTR (black) and DSS (red) mice in the left hippocampus and left somatosensory cortex, respectively. The boxes range from the 25% and the 75% percentiles; the 5% and 95% percentiles are indicated as error bars; single data points are depicted as full circles. Medians are represented by horizontal lines within each box. In Panel 6C for Cr, PCr, Cr+PCr, GPC+PCh, Glu, Glu+Gln, Tau, and Glyc *n* = 14 CTR and *n* = 17 DSS‐treated mice were considered. In Panel 6F for Cr, PCr, Cr+PCr, GPC+PCh, Glu, Glu+Gln, Tau, and Glyc *n* = 16 CTR and *n* = 14 DSS‐treated mice, and for Cr *N* = 16 CTR and *N* = 13 DSS‐treated mice were considered. Statistical significance was retrieved from an unpaired Student *t* test (two‐tailed) and indicated with the asterisks (*P < 0.05, **P < 0.01, ***P < 0.001, ****P < 0.0001 vs. control). Cr, creatine; CTR, control; DSS, dextran sulfate sodium; Gln, glutamate + glutamine; Glu, glutamate; Glyc, glycine; GPC, glycophosphorylcholine; PCh, phosphorylcholine; PCr, phosphocreatine; Tau, taurine

Based on our data showing impairment in astrocyte–neuron contacts, we then investigated the impact on brain energetic supplementation by using proton MRS (^1^H MRS), a useful tool to examine neurochemical changes in the brain, as it provides information about the local concentration of metabolites in tissues (Figure 7C–F). In both the hippocampus (Figure 7A) and cortex (Figure 7D) of DSS‐treated mice, a significant average reduction of total choline (GPC+PCh; −30% in the hippocampus, −43% in the cortex), glutamate (Glu; −9% in the hippocampus, −10% in the cortex), Glu+glutamine (Gln; −5% in the hippocampus, −7% in the cortex), and taurine (Tau; −10% in the hippocampus, −12% in the cortex) and an average increase of glycine (Gly; +14% in the hippocampus, +15% in the cortex) were detected resorting to the spectra analysis with LCModel; major alterations were also detectable by visual inspection (Figure 7B–E). In addition, in DSS‐treated mice a decrease of GABA was observed in the hippocampus (−12%) and of alanine (Ala; −21%), aspartate (Asp; −21%), N‐acetyl‐aspartate (NAA)+N‐acetyl‐aspartyl‐glutamate (NAAG; −4%), and several macromolecules (MM) and lipids in the cortex. A slight trend to increase of TCr (+3%) and a strong imbalance of the creatine/phosphocreatine (Cr/PCr) ratio, with a substantial raise of PCr (+20% in the hippocampus, +15% in the cortex) at the expense of Cr (−18% in the hippocampus, −20% in the cortex), was also observed in the DSS‐treated mice. In this regard, however, it must be remembered that, despite the strong Cr/PCr alterations in DSS mice being repeatedly detected in different regions, the concentration of the single metabolite, Cr or PCr, in the framework of short‐TE ^1^H MRS is intrinsically highly correlated to the other one, due to their strong spectral overlap.

The ^1^H MRS analysis indicated a consistent and general trend of reduction of the brain metabolite and macromolecule concentration in DSS‐treated mice, providing evidence of diffuse biochemical alterations, tentatively ascribable to an impairment of the metabolic supply and clearance mechanisms. Complete data reporting for all metabolite and macromolecule concentrations is available (extended data table for cortex and hippocampus).

## 4 DISCUSSION

Our data show how a single inflammatory trigger in the gut can drive significant changes in the CNS, disrupting the circadian clock machinery and brain fluid distribution, leading to impairment of glymphatic clearance and altered neurotransmitter release dynamics. These ultimately impact brain function, extending to sensory and behavioral regulation, in line with previous evidence.^43^, ^44^, ^45^ Due to its high molecular weight and hydrophilic nature, DSS does not cross the BBB;^15^ thus, any central effects observed in this model result from systemic inflammation and gut–brain axis dysfunction and cannot be attributed to direct exposure of the brain to DSS.

Accordingly, after colonic DSS challenge in mice, we observed that the misalignment of the local circadian clockwork is mirrored at the central level. The loss of circadian clock components has been related to IBD pathogenesis, confirming that circadian clock genes are key regulators of colitis.^14^, ^36^ Our results are in line with data from literature on young, newly diagnosed, untreated patients with IBD (ClinicalTrials.gov Identifier: NCT03662646),^46^ as well as on patients with active UC.^47^ Consistently, other studies demonstrated that colitis severity increased after disrupting the circadian clock, including the genes *Per1/2* and *Nr1d1*.^36^, ^48^, ^49^ On the other hand, a desynchronization of primary and subordinate brain oscillators is related to neuroinflammation.^50^ *Rev‐erbα*, significantly reduced in brain regions in our experimental setting, has been reported as a crucial modulator of neuroinflammation.^51^ Inflammatory pathways undergo parallel changes in the periphery and in the CNS, while the peripheral and central clockworks show a general opposite trend, suggesting a desynchronization of the central clock versus the peripheral one.

These results are compelling, as the regulation and efficiency of brain fluid dynamics are under circadian regulation.^12^ It is noteworthy that the loss of AQP4 eliminates the day–night differences in both brain fluid influx and drainage.^12^ Our results provide valuable insight into the comprehension of CNS response to acute colitis, defining the central role of the clock gene machinery. Previously, few studies have attempted to relate chronic colitis to glymphatic dysfunction; however, neuroinflammation has been considered the only colitis‐induced exacerbating factor among the driving mechanisms.^8^, ^52^

Our results of MRI analysis show a significant increase of SE in DSS‐treated mice; this alteration is related to an accumulation of gadolinium‐based contrast agent in perivascular spaces.^53^ This highlights an altered interchange between CSF in the perivascular compartment and ISF in the parenchymal compartment, namely a defect in glymphatic circulation. To further explore the alteration of brain solute movement, we performed a proteomic analysis of CSF under colitis conditions, revealing substantial differences in protein profiles. First, we observed in DSS‐treated mice an upregulated expression of factors related to inflammatory pathways (ORM1, PGLYRP1, CTLA2A, SERPINA3N, SERPINA3M), proving a transfer of an inflammatory condition from periphery to CNS through signals within the circulating fluids. Factors related to the immune and complement response were also differentially expressed in the CSF of DSS animals, suggesting that the immune surveillance system also plays a role in regulating peripheral‐central signaling (upregulated in DSS: *FGL1, ORM2, PGLYRP1, HPX, LCN2, CTLA2A*; downregulated in DSS: *IGHV4‐1, IGHG2B, APOA4, IIGHG1, IGHG3, C8G, IGKV6‐13, IGHV6‐6, IGKV1‐117, IGHV1‐54, IGKV4‐55, IGKV12‐46, LAG3*). Moreover, the presence of both visceral and referred somatic pain in our animal model, due to somatosensory cortex integration of spinothalamic projections of visceral afferents,^54^ may lead to enhanced mobilization and/or retention of pain‐related soluble factors. These molecules, traveling through CSF, may contribute to the dysregulation of solute trafficking across brain fluid compartments. Accordingly, by proteomic analysis on the CSF, we observed in DSS mice the upregulation of CACNA2D1, SERPINA3N, and LRP1 and the downregulation of ANXA2, mediators involved in pain signaling.^55^, ^56^, ^57^, ^58^ The deregulation of factors involved in the maintenance of brain cholesterol homeostasis (APOA1, APOA4, APOA2, SAA1, NPC2, LRP1) was also detected. Brain cholesterol is not only an essential structural component but also a molecule required for synapse and dendrite formation as well as axonal guidance. Cholesterol depletion in neurons impairs synaptic vesicle exocytosis, neuronal activity, and neurotransmission, leading to synapse degeneration.^59^ In this context, we also observed an upregulation of L1CAM in the CSF under DSS conditions. This adhesion molecule, expressed during the development of both the central and peripheral nervous systems, plays critical roles in neuronal migration, axon growth and guidance, fasciculation, and synaptic plasticity. This upregulation may result from altered expression or increased shedding into the CSF,^60^ possibly correlating with poor neuronal connections. In addition, neuronal deletion of L1CAM has been found to accelerate the age‐related decline in hippocampal neurogenesis,^61^ thus suggesting the existence of compensating neurogenesis potentially associated with brain ventricle enlargement.^62^ Finally, the downregulation of the glial structural protein GFAP in the CSF of DSS‐treated mice further underscores the strong involvement of astrocytes, a pivotal cellular component of the glymphatic system.

The conclusion of impaired glymphatic transport is supported by data showing preserved BBB function in our experimental model, suggesting maintained vascular integrity despite compromised clearance efficiency. This dysfunction leads to cerebral accumulation of waste products, such as phosphorylated tau and amyloid oligomers. These results align with data showing that DSS‐mediated intestinal inflammation is correlated with altered amyloid deposition and glial immunoreactivity in the mouse brain.^63^ The impaired control of glymphatic clearance, in turn, promoted an increase in ventricle volume, a condition associated with brain suffering, related to functional and morphological alterations of the choroid plexus.^64^ Notably, ventricle enlargement and the upregulation of functional pathways primarily related to neuroinflammation and cell‐to‐cell interactions appear to be interdependent, as demonstrated by evidence from neurological and psychiatric studies in humans.^65^, ^66^ Notably, changes in choroid plexus volume and permeability have been linked to inflammatory activity in CD patients, thus supporting the idea that intestinal inflammation may influence brain fluid dynamics.^67^

Glymphatic solute transport and fluid flow are specifically mediated by astrocytes, which regulate ISF balance by reabsorbing osmolytes and ions and facilitating water movement, specifically through the activity of AQP4.^68^ In our experimental model, the observed reduction in astrocytic markers and AQP4 protein expression suggests impaired astrocyte function in the brain. This correlated with an altered metabolic activity in the cortex and hippocampus, matched with an unbalanced release of neurotransmitters in the synapses. These areas are involved in the behavioral alterations characterizing the model, including visceral hypersensitivity, somatosensory alterations (thermal hyperalgesia, mechanical and thermal allodynia), and mood disorders, such as anxiety‐related behaviors. The cortex is a key component of the pain matrix,^69^ while the hippocampus and the associated limbic system play crucial roles in emotional processes and mood regulation.^70^ Notably, these two regions are early targets in neurodegenerative diseases, such as AD and PD,^71^ and in our model, they exhibit increased waste accumulation. In turn, we found specific changes in astrocyte‐to‐neuron communication, resulting from disruptions in the tetra–partite synapse and in brain energy supply, as shown by MRS data. In particular, the prevalence of glutamatergic exocytosis efficiency from astrocyte specializations over the glutamatergic signal at the “pre to post” synaptic interface resulted in the prevailing of the local astrocyte‐to‐neuron crosstalk over the neuronal “long‐distance” transmission.^72^ This imbalance suggests a synaptic impairment, potentially supporting an impaired ability to activate resilience circuits aimed to correct functional and behavioral alterations, as also sustained by the anxiety‐like behavior observed in DSS‐treated mice. This view is consistent with the reduced glutamate recovery mechanism (particularly at the cortical level) and also with the painful hypersensitivity that characterizes the model, as well as the clinical condition of IBD.^73^

Neuronal hyperactivity contributes per se to the chemical composition of the ISF;^74^ circularly, it also results from the glymphatic imbalance demonstrated in our study, which ultimately affects synaptic function by promoting the accumulation of waste products, exacerbating neuronal hyperexcitability, and compromising excitatory/inhibitory balance.

These data provide, for the first time, a detailed characterization of the pathogenic association between colonic acute inflammation and disruption in the clockwork machinery associated with glymphatic dysfunction. This impairment leads to an imbalance of fluid and solute homeostasis within brain tissue, contributing to the onset of synaptopathy and, consequently, to somatosensory and mood disorders. The characterization of the cascade of events and the relationship among gut inflammation, brain fluid dynamics alteration, and brain waste product deposition provides insight into how a peripheral enteric hit may disorganize the central hierarchy, potentially triggering a prodromal process that, if persistent, may culminate in neurodegeneration. Our results highlight the importance of the glymphatic system in the centralization of peripheral alterations induced by inflammatory colitis, also supporting the onset of CNS‐related comorbidities. Moreover, they outline the restoration of glymphatic homeostasis as a target to direct pharmacological research efforts in the context of IBD. Furthermore, from a translational perspective, understanding which types of inflammatory insults may derange the daily rhythm of the mechanisms controlling brain waste disposal is relevant, as it may help define specific at‐risk populations with increased susceptibility to neurological disorders.

## CONFLICTS OF INTEREST STATEMENT

The authors declare no competing interests. Author disclosures are available in the supporting information.

## CONSENT STATEMENT

No human subjects were included in the study. Consent was not necessary.

## Supporting information



Supporting Information

Supporting Information
