# Supplementary material for: Intestinal inflammation induces glymphatic remodeling, priming early neurodegenerative signals in male mice
Source: Alzheimers Dement. 2025 Oct 9;21(10):e70640. doi: 10.1002/alz.70640 (PMC12509038; doi:10.1002/alz.70640)
Supplement: Supplementary file 2 — Supporting Information [file ALZ-21-e70640-s002.docx]

**List of Supplementary Materials**

Supplementary materials and methods

Supplementary references

Supplementary figures and figure legends 1-5

**Supplementary materials and methods**

**Reagents**

Dextran sulfate sodium salt was purchased from CliniSciences (Italy, Guidonia Montecelio). Gadovist 1M was purchased from Bayer. Gadovist 20 mM was obtained by diluition in artificial CSF (NaCl 299,5 mM; KCl 6 mM; CaCl_2_ * 2 H_2_O 2,8 mM; MgCl_2_ * 6 H_2_O 1,6 mM; NaH_2_PO4 * H_2_O 0.39 mM; Na_2_HPO_4_ * 2 H_2_O 1.60 mM). For MRI analysis, anesthesia was induced in animals by intraperitoneal injection of a mixture of ketamine 80 mg/kg (Lobotor) and xylazine 8 mg/kg (Sedaxylan) and maintained by continuous subcutaneous administration of ketamine 40 mg/kg and xylazine 4 mg/kg. For MRS experiments, anesthesia was induced with a mixture of O_2_/isoflurane (Iso-Vet 1000 mg/g, Piramal Critical Care B.V.). Antibodies used are detailed as supplementary data (extended data table antibody).

**Assessment of Visceral Sensitivity by Visceromotor Response (VMR)**

Visceromotor response (VMR) to colorectal distension (CRD) was performed according to a previously described method (1) with minor changes. One week before the beginning of treatments, two electromyographic (EMG) electrodes (AS631, Cooner Wire, Chatsworth, CA, USA) were sutured into the external oblique abdominal muscle and exteriorized dorsally in the animals under anesthesia (2% isoflurane). To perform the test, a lubricated latex balloon (2 cm) assembled into an embolectomy catheter, attached to polyethylene tubing, and connected to a syringe filled with water, was inserted through the anus into the rectum and descending colon of the mice anesthetized with isoflurane (2% isoflurane). The tubing was taped to the tail in order to hold the balloon in place. Mice were then allowed to recover from anesthesia for 30 min. CRD was performed by filling the syringe with increasing volumes of water (50, 100, 150 and 200 µL). During the measure, the electrodes were connected to a data acquisition system, and the corresponding EMG signals were recorded, amplified, filtered (Animal Bio Amp, ADInstruments, Colorado Springs, CO, USA), digitized (PowerLab 4/35, ADInstruments), analyzed, and quantified using LabChart 8 (ADInstruments). To quantify the magnitude of VMR at each distension volume, the area under the curve (AUC) immediately before distension (30 s) was subtracted from the AUC during balloon distension (30 s), and the responses were expressed as a percentage increase from the baseline. The time elapsed between two consecutive distensions was 5 min.

**Assessment of Visceral Sensitivity by Abdominal Withdrawal Reflex (AWR)**

Abdominal Withdrawal Reflex (AWR) to colorectal distension (CRD) was assessed by using a semiquantitative score (1). To perform the test, a lubricated latex balloon (2 cm) assembled into an embolectomy catheter, attached to polyethylene tubing, and connected to a syringe filled with water, was inserted through the anus into the rectum and descending colon of the mice anesthetized with isoflurane (2% isoflurane). The tubing was taped to the tail in order to hold the balloon in place. The mice were then allowed to recover from anesthesia for 30 min. AWR measurement consisted of visual observation of animal responses to graded CRD (50, 100, 150 and 200 µl) by blinded observers who assigned an AWR score: no behavioral response to CRD (0); immobility during CRD and occasional head clenching at stimulus onset (1) mild contraction of the abdominal muscles but no abdominal lifting from the platform; (2) strong contraction of the abdominal muscles and lifting of the abdomen off the platform (3); arching of the body and lifting of the pelvic structures and scrotum (4). The time elapsed between two consecutive distensions was 5 min.

**Assessment of thermal allodynia by Cold plate test**

Thermal allodynia was assessed using the Cold plate test. With minimal animal–handler interaction, mice were taken from home cages and placed onto the surface of the cold plate (Ugo Basile, Varese, Italy) maintained at a constant temperature of 4°C ± 1°C. Ambulation was restricted by a cylindrical Plexiglas chamber (diameter, 10 cm; height, 15 cm) with an open top. A timer controlled by a foot switch was used to monitor timing response latency from the moment the mouse was placed onto the cold plate. Pain-related behavior (licking of the hind paw) was observed, and the time (seconds) of the first sign was recorded. The cutoff time of the latency of paw lifting or licking was set at 30 s (2). Sample size was CTR n=9, DSS n=10.

**Assessment of thermal hyperalgesia by Hot plate test**

Thermal hyperalgesia was assessed using the Hot plate test. With minimal animal–handler interaction, mice were taken from home cages and placed onto the surface of the cold plate (Ugo Basile, Varese, Italy) maintained at a constant temperature of 45°C ± 1°C. Ambulation was restricted by a cylindrical Plexiglas chamber (diameter, 10 cm; height, 15 cm) with an open top. A timer controlled by a foot switch was used to monitor timing response latency from the moment the mouse was placed onto the cold plate. Pain-related behavior (licking of the hind paw) was observed, and the time (seconds) of the first sign was recorded. The cutoff time of the latency of paw lifting or licking was set at 30 s. Sample size was CTR n=9, DSS n=9. One DSS-treated animal was excluded from the analysis because it was non-responder to the test and therefore not assessable.

**Assessment of mechanical allodynia by von Frey test**

The animals were placed in 20 × 20 cm Plexiglas boxes equipped with a metallic mesh floor, 20 cm above the bench. A habituation of 15 min was allowed before the test. An electronic von Frey hair unit (Ugo Basile, Varese, Italy) was used, and the withdrawal threshold was evaluated by applying force ranging from 0 to 5 g with a 0.2 g accuracy. Punctuate stimulus was applied to the mid-plantar area of each anterior paw from below the meshy floor through a plastic tip, and the withdrawal threshold was automatically displayed on the screen. The paw sensitivity threshold was defined as the minimum pressure required to elicit a robust and immediate withdrawal reflex of the paw. Voluntary movements associated with locomotion were not taken as a withdrawal response. Stimuli were applied on each anterior paw with an interval of 5 s (2). Sample size was CTR n=8, DSS n=8. One CTR animal and two DSS-treated animals were excluded from the analysis because they were non-responders to the test and therefore not assessable.

**Animal preparation for *in vivo* imaging**

For Magnetic Resonance Imaging (MRI) scans, mice were anesthetized with a mixture of ketamine/xylazine (i.p.). Before positioning in the MRI magnet, animals underwent an injection of 10 μL of a 20mM Gadovist solution (Bayer AG) in the cisterna magna at a rate of 1 μL/min for a total of 10 minutes, by means of a microinjector (Quintessential Sterotaxic Injector, Product N° 53311, Stoelting, supplied by 2Biol Italy) and were subsequently placed in prone position on a heated bed equipped with a mask, for continuous O_2_ supply, and ear and bite bars to immobilize the head. During the imaging session i.p. ketamine/xylazine anesthesia was supplied by means of an infusion pump and the respiratory rate, measured by means of a pneumatic pillow placed under the abdomen, was kept around 90-120 bpm.

For Magnetic Resonance Spectroscopy (MRS) experiments, mice were anesthetized with a mixture of O_2_/isoflurane (4% for induction) and subsequently placed in prone position on the same bed used for MRI. During the MRS sessions, the flux of O_2_/isoflurane was lowered to 1 L/min at 1-2% to attain a respiratory rate of 40-70 breaths/min. Each experimental session was run to obtain either a ^1^H localized MRS spectrum of the left hippocampus (N=17 CTR, N=19 DSS) or a ^1^H localized MRS spectrum of the left somatosensory cortex (N=16 CTR, N=16 DSS) separately and lasted approximately 1-1.5 hours.

At the end of the imaging sessions, mice were extracted from the magnet and sacrificed to collect tissues for subsequent analyses.

**MRI/MRS acquisition**

The *in vivo* MRI/MRS scans were performed using a 7 Tesla Bruker Pharmascan 70/16US, equipped with a high-power gradient insert (760 mT/m maximum amplitude). A 72 mm inner diameter circularly polarized volume coil was used for radiofrequency transmission, whereas a circularly polarized surface coil suited for the mouse head was used for reception.

For contrast enhanced MRI, after rapid positioning in the center of the magnet and optimization of basic MRI parameters, mice were repeatedly scanned with a High-resolution 3D T1-weighted imaging Fast Low Angle Shot (FLASH) sequence allowing to obtain one sagittal dataset of the whole brain every 6 min 26 s which was rounded to 6 min for the statistical analysis (Repetition time (TR)=25 ms, Echo Time (TE)=5.5 ms, Flip Angle (FA)=25°, field of view (FOV)=17 x 15 x 15 mm^3^ (Read x Phase x Slice), read direction rostro-caudal, matrix (MTX)=154 × 136 × 136 yielding 110.4 × 110.3 × 110.3 µm^3^ resolution). The procedure timing was standardized across the animals (N=12 CTR, N=14 DSS): considering the initial transfer of the animal inside the scanner, the experiment allowed to consistently monitor the contrast agent dynamics starting from 25-31 minutes after the end of infusion, for a period of 50 min - 1 hour. Postponed start either anticipated end of the MRI acquisition for some animals led to a variation of the statistical group size in the early and late time points respectively. This was accounted for with suitable statistical corrections (see section on Gadolinium enhancement quantification**)** in order to analyze the whole experimental time course. The 3D T1 FLASH acquisition was performed also in a small group of sham mice (N=4), not injected with contrast agents, in order to estimate the baseline MRI signal of the brain with the same sequence.

For ^1^H MRS two 2D Rapid Acquisition with Relaxation Enhancement (RARE) T_2_ weighted anatomic datasets were acquired for precise MRS voxel positioning (TR=2500 ms, TE=33 ms, FOV=20 x 20 mm, MTX=256 × 256, Slice Thickness (ST)=0.7 mm, 9 slices, 2 averages, RARE factor=8, axial and coronal slice orientation). Afterwards, for ^1^H MRS of the hippocampus, a Point Resolved Spectroscopy (PRESS) protocol (TR=2500 ms, TE= 16.5 ms, 640 averages, spectral width 11.1 ppm, 2048 points) equipped with the VAPOR water suppression, the outer volume suppression, reference and navigator scans of water, was acquired in a 1.8 x 1.1 x 2 mm^3^ oblique voxel positioned in the left lobe (3.96 µL volume). Alternatively, for ^1^H MRS of the somatosensory cortex, a PRESS protocol was acquired with the same parameters in a 1 x 1.7 x 2.5 mm^3^ oblique voxel (4.25 µL volume) positioned in the left lobe.

Before MRS spectra acquisition, the unsuppressed water linewidth in the voxel was adjusted by means of an automatic 1^st^ and 2^nd^ order Local Shimming with the Bruker MAPSHIM utility, based on the B_0_ map of the brain acquired *in vivo* during the experimental session. In the hippocampus a linewidth in the range of 9-15 Hz while in the somatosensory cortex a range of 12-16 Hz were attained respectively. To avoid recurring failure in the MAPSHIM local shimming calculations, a global 1^st^ and 2^nd^ order study shimming based on the STEAM sequence was performed before the MRS acquisition in the somatosensory area, owing to its position closer to the skull.

MRS spectra reconstruction was performed applying eddy current correction and retrospective field drift compensation algorithms. Before subsequent evaluations, spectra underwent visual inspection. Few spectra corrupted by external lipid signal contamination, characterized by deformed baselines, poor water suppression or acquired during instrumental instabilities were excluded from further analyses.

^1^H MRS scans were performed without water suppression in N=2 CTR in both hippocampus and cortex, in N=2 DSS mice in the hippocampus and other N=2 DSS mice in the cortex to measure *in vivo* the spin-lattice relaxation time (T1) and the spin-spin relaxation time (T2) of water in the voxel. T1 was estimated by means of an Inversion Recovery (IR) acquisition, applying an hyperbolic secant inversion pulse of 5 ms before the PRESS sequence with fixed TE=16.5 ms, varying the Inversion Time (TI) between 52 ms and 20 s. T2 was estimated varying the TE of the PRESS sequence between 16.5 and 250 ms, keeping a fixed TR=10 s. In 1 DSS mouse T1 could not be measured in the hippocampus due to a failure in the setting of the IR sequence.

**Gadolinium enhancement quantification**

Gadolinium-perfused brain images are characterized by a signal intensity gradient (higher signal in the dorsal regions) due to the usage of a surface coil for reception.

Sample size for each time point was CTR n= 9 for 25’, n=10 for 31’, 37’, 43’, 49’, 55’, 61’; n=11 for 67’, 73’; n=5 for 79’; DSS n=12 for 25’, 31’; n=13 for 37’, 43’, 49’, 55’, 61’, 67’; n=12 for 73’; n=4 for 79’.

An intensity enhancement analysis was conducted on a manual selected ROI based on the hyperintense areas marking the presence of gadolinium-based contrast agent. This area represents the perivascular route of the CSF at the level of the large cerebral blood vessels at the base of the skull up to the olfactory bulb. For the selection of the ROI and the analysis of the mean intensity, the program 3D Slicer was used, monitoring the variation of the signal over time, manually aligning the images where necessary. The data are expressed as the percentage signal enhancement ratio to baseline. Sample size for each time point include C CTR n= 9 for 25’, n=10 for 31’, 37’, 43’, 49’, 55’, 61’; n=11 for 67’, 73’; n=5 for 79’; DSS n=12 for 25’, 31’; n=13 for 37’, 43’, 49’, 55’, 61’, 67’; n=12 for 73’; n=4 for 79’. A control animal and a DSS-treated animal that showed excessive enlargement of the cerebral ventricles were excluded from the analysis.

Extraction of the lateral ventricles volume was performed manually using the Medical Image Processing, Analysis, and Visualization (MIPAV) software. Segmentation was performed by manually drawing a volume of interest (VOI) for the right and left lateral ventricles, separately. For each group the last time point was considered and one mouse/group was excluded due to an enormous increase in the ventricles volume (n=11 controls; 13 DSS).

**MRS spectra quantification**

^1^H-MRS spectra were analyzed using LCModel ver 6.3 1-R *(3)*. For all spectra the simulated model basis provided by the software developer for 7 Tesla acquisitions [http://s-provencher.com/lcm-basis.shtml] was used, including metabolites, macromolecules (MM) and Lipids (Lip): alanine (Ala), aspartate (Asp), creatine (Cr), γ-aminobutyric acid (GABA), glucose (Glc), glutamate (Glu), glycine (Glyc), glutamine (Gln), glutathione (GSH), glycophosphorylcholine (GPC), myo-Inositol (Ins), lactate (Lac), N-acetyl-aspartate (NAA), N-acetyl-aspartyl glutamate (NAAG), phosphorylcholine (PCh), phosphoethanolamine (PE), scyllo-inositol (Scy), taurine (Tau), MM09 (at 0.9 ppm), MM12 (at 1.2 ppm), MM14 (at 1.4 ppm), MM17 (at 1.7 ppm), MM20 (at 2 ppm), Lip09 (at 0.9 ppm), Lip13a and Lip13b (at 1.3 ppm), Lip20 (at 2 ppm) and also took into account a negative correction for the fit of the Cr-CH2 signal. The software was run in the 4.1-0.2 ppm window with default soft constraints and prior knowledge on metabolite ratios, except for Tau being higher in rodents, and fixing the Spline baseline knot spacing (DKNTMN) to 0.1. Water scaling was enabled, setting water concentration *wconc*=43300 mmol/kg, and relaxation attenuation factors *ATTH2O* and *ATTMET* were set equal to 1 in view of subsequent relaxation corrections. The reliability and quality of the fitting procedure was assessed by visual inspection and a quality threshold was considered: only spectra with signal to noise ratio (SNR)>9 and Full Width at Half Maximum of metabolites (FWHM) <0.044 ppm (integer SNR and FWHM calculated by LCModel) were retained. Also, the presence of distortions of the lineshape not handled by LCModel were considered as a criterion for exclusion. Definitely, on the basis of the aforementioned quality assessment principles, 1 spectrum was excluded for lipid contamination, 3 spectra for having SNR<10, 1 spectrum for distortions in the lineshapes, 2 for instrumental instabilities, yielding a final group size of N=14 CTR, N=17 DSS for the hippocampus MRS and N=16 CTR, N=14 DSS for the somatosensory cortex MRS. The final average quality parameters of the spectra were: SNR=14.1 (SD=1.9) and FWHM=0.025 ppm (SD=0.005 ppm) for the hippocampus, SNR=13.4 (SD=1.8) and FWHM=0.030 ppm (SD=0.006 ppm) for the cortex.

Metabolite concentrations with Cramér-Rao lower bounds (CRLB, an estimate of the lower limit of the standard deviation) lower than 50%, were considered for further analysis, as reported in other works to avoid over-filtering of the data *(4, 5)*. For subsequent analyses metabolites estimated in almost N=12 animals were taken into account. In case of highly overlapping spectra, the sum of the concentrations was also calculated: total creatine (tCr) = Cr + PCr and total choline (tCho) = PCh + GPC. Infact, the quantification of the sum is indeed more reliable and characterized by a very low CRLB (for Cr+PCr around 2%), while the quantifications of the single metabolites, Cr either PCr, PCh either GPC, in the framework of short-TE ^1^H MRS are intrinsically highly correlated.

For the water T1 and T2 estimate in the MRS voxels, integrals of the magnitude spectra of the water signal were obtained with Bruker Topspin, after the baseline correction. Water T1 was calculated fitting the integrals with Matlab R2023b as a function of TI to the recovery law I(t)=I0*|1-2*a*(exp(-t/T1)| where I0 represents the equilibrium value of the integral and a is a factor that takes into account imperfect inversion. Water T2 were calculated fitting the integrals as a function of TE to the single exponential decay I(t)=I0*exp(-t/T2). The average T1 and T2 values obtained in all the animals were set as reference effective water relaxation times in the gray matter rich voxels set in this study for subsequent quantifications: T1_H2OGM_=1.39 s, SD=0.05 s (N=7); T2_H2OGM_=46.66 ms, SD=2.15 ms (N=8).

**Scaling of metabolite concentration in mmol/L**

The LCModel results were not reported as ratios over total creatine (tCr, Cr+PCr), as it is often found in the literature, but rather as concentrations (mmol/L), owing to a slight trend to increase of tCr (+3%) and a strong unbalance of the Cr/PCr ratio (Fig.6). Concentrations of metabolites (mM) were obtained under the assumption of pure gray matter voxels, resorting to the LCModel output values expressed in institutional units. The formula and the parameters used for the conversion to mM units are based on well-known methods (see for example *(6)*). Considering a pure gray matter voxel, i.e. having voxel fractions of gray matter f_GM_=1, white matter f_WM_=0 and CSF f_CSF_=0, the following formula was applied to obtain metabolite concentrations *M* in mmol/L:

$$M= M_{LCM}*\rho*\frac{R_{H2OGM}}{R_{MGM}}$$

where $\rho$ is the water density at 37° and 1 atm, $M_{LCM}$is the LCModel output and $R_{H20GM}$ and $R_{MGM}$ are the relaxation weighting factors for water and metabolites. $M_{LCM}$considers scaling of measured metabolite signals $S_{Mobs}$ over the measured water signal $S_{H2Oobs}$, assuming molal concentration in gray matter *wconc* and the number of protons for each compound *N_M_* according to:

$M_{LCM}= \frac{S_{Mobs}}{S_{H2Oobs}}*\frac{2}{N_{M}}*wconc.$

The relaxation weighting factors for water and metabolites are:

$R_{H20GM}=exp(-TE/{T2}_{H2OGM})*[1-exp(-TR/{T1}_{H2OGM})]$,

$R_{MGM}=exp(-TE/{T2}_{MGM})*[1-exp(-TR/{T1}_{MGM})]$.

Concentrations of macromolecules and lipids were obtained by taking into account the water relaxation times correction factors only.

This approximation could introduce an overestimation of the concentrations in presence of a sizable CSF and white matter fraction in the MRS voxel. However, voxel positioning prior acquisition was accurately controlled to avoid the inclusion of CSF, in particular in the case of hippocampus MRS, as one can notice in Fig. 7A-7D. Gray matter is indeed the major component of the MRS voxels. In absence of a precise volumetric estimate of the voxel composition for each animal, a maximum fraction of white matter up to 35% could be reasonably estimated in the hippocampus voxel on the basis of the T2-RARE images, while in the somatosensory cortex voxel the gray matter prevalence is evidently stronger.

The metabolite relaxation weighting factors used to retrieve metabolite concentrations have been calculated on the basis of literature data in both humans and rodents for gray matter at 7 Tesla, detailed in the extended data Table (water and metabolite relaxation times).

**Proteomic analysis**

CSF samples (20 µl) collected from each mouse were loaded on the washed EN-BEADS (25 µl of EN-BEADS suspension used for each sample) of the ENRICH-iST kit (PreOmics, Germany), and added with 80 µl of EN-BIND buffer (provided by the kit). After an incubation on thermoshaker at 30 ºC for 30 minutes at 1,200 rpm, the magnetic beads were washed three times using 100 µl of EN-BIND buffer. The enriched fraction was added with 50 µl of LYSE-BCT and incubated at 95 °C for 10 minutes at 1,200 rpm. Protein digestion was carried out using trypsin (provided by the kit) with a 3-hour incubation at 37 °C. After adding 100 µl of STOP solution, the peptides were desalted using the CARTRIDGE and the solvents provided by the kit. The purified peptides were dried under vacuum and then dissolved in 11 µl of LC-LOAD buffer provided by the kit for mass spectrometry-based analysis. Peptide mixtures were injected in a nano LC system (EASY-nLC 1000 Integrated Ultra High Pressure Nano-HPLC System, Proxeon Biosystem) for separation on a 75 µm i.d. x 15 cm reverse-phase silica capillary column, packed with 1.9 µm ReproSil-Pur 120 Å C18-AQ (Dr. Maisch GmbH, Germany). A 100-minute gradient of eluents A (pure water with 0.1% v/v formic acid) and B (acetonitrile with 0.1% v/v formic acid) was used for peptides separation (from 5% to 40% of B in 88 min, 300 nl/min flow rate). Mass and tandem mass spectra were acquired using a Q-Exactive mass spectrometer (Thermo Scientific, Bremen, Germany) equipped with a nano-electrospray ion source (Proxeon Biosystems). Due to the low amount of material, each sample was analyzed in single technical replicate. Full scan spectra were acquired with the lock-mass option, resolution set to 70,000 and mass range from *m/z* 300 to 2000. The ten most intense ions (charge exclusion: unassigned, 1, 6–8, >8) were selected for fragmentation (ddMS2). Tandem mass spectra were acquired at a resolution of 17,500, with NCE set to 25 and an isolation window of 2 *m/z*. For data normalization, peptide concentration in the eluted fractions from the kit were measured using the Pierce Quantitative Colorimetric Peptide Assay (Thermo Scientific), following the manufacturer's instructions. The sample with the lowest peptide concentration was used as a reference, and intensities were adjusted accordingly to ensure consistent nLC-MS/MS signal levels across all samples. Raw mass spectra were then processed using MaxQuant software (v. 1.6.1.0) *(7)* for label-free protein quantification based on the precursor intensity, using the following parameters: trypsin as proteolytic enzyme; 2 missed cleavages allowed; carbamidomethylation on cysteine residues as fixed modification; protein N-terminus-acetylation and methionine oxidation as variable modifications; mass tolerance was set to 5 ppm and to 20 ppm for precursor and fragment ions, respectively. FDR of 1% was selected for both peptide and protein identification, with minimum two peptides per protein and at least one unique. The full dataset of identified and quantified proteins was further analyzed using a custom script developed in the R programming language. The mass spectrometry proteomics data have been deposited to the ProteomeXchange Consortium via the PRIDE *(8)*.

**Tissue collection**

The colon tissues were harvested, measured, and numbered separately. The tissues were quickly rinsed with phosphate-buffered saline (PBS) to remove lumen contents, freeze-dried, and then stored at –80°C to be used later in quantitative polymerase chain reaction (qPCR) and Western blot. Distal colon segments were prepared for hematoxylin and eosin (H&E) staining. Brain areas (hypothalamus, hippocampus and cortex), once isolated, were freeze-dried, and then stored at –80°C for following determinations.

**RNA extraction and Real Time – Quantitative Polymerase Chain Reaction (RT-qPCR)**

RNA extraction was executed on tissues obtained by the processing of brain areas and colons isolated from DSS and control mice.

Total RNA extraction of brain tissues was performed through RNeasy® Mini Kit (ID: 74104, Qiagen, Hilden, Germany) following the manufacturer’s instructions. The extracted RNA was quantified using the Quantifluor^®^ RNA System Kit (Promega), and then reverse transcribed using the QuantiTect^®^ Reverse Transcription kit (205313, Qiagen, Hilden, Germany), according to the manufacturer’s instructions.

Total RNA extraction from the colon was performed using PureZOL RNA Isolation Reagent (Bio-Rad Laboratories, USA, Cat #7326890) and extracted using a NucleoSpin kit (Macherey-Nagel, DEU, Cat #FC140955N) according to the manufacturer’s instructions. RNA quality and quantity were determined using a NanoDrop spectrophotometer (Thermo Fisher Scientific, USA). cDNA was obtained using a High-Capacity Reverse Transcription Kit (Thermo Fisher Scientific, USA, Cat #4374966). The complementary DNA was then analyzed by RT-qPCR through the use of the QuantiTect® SYBR Green PCR Kit (Qiagen, Hilden, Germany) and the Rotor-Gene Q plex HRM System (Cat. No. 9001650 (Qiagen, Hilden, Germany) for brain-derived cDNAs and a Bio-Rad CFX96 Connect Real-time PCR System instrument and software for colon cDNAs. The relative mRNA levels were normalized against glyceraldehyde-3-phosphate dehydrogenase (*Gapdh*) or β-actin (*Actb*). The quantification of the amplified transcripts was performed by the 2^-ΔΔCT^ method. The primers used for the analyses were provided by Qiagen (Hilden, Germany) and are reported as supplementary information data (extended data table primers).

**Biochemical evaluations of serum inflammatory/immune mediators**

Blood was collected from all experimental groups at the end of the experimental protocol. Then, serum was obtained by centrifugation at 2500 rpm at 4 ◦C for 12 min and stored at -80◦C for subsequent biochemical analyses. The concentration of twenty-three pro- and anti-inflammatory mediators and factors of innate and adaptive immunity were obtained by a high-sensitivity kit Bio-Plex Pro Mouse Cytokine 23- plex (cod. M60009RDPD) using the Bio-Plex 200 System and Luminex xMAP technology (Bio-Rad Laboratories, Inc., USA). This multiplex assay detects: IL-1𝜶, IL -1β, IL -2, IL -3, IL -4, IL -5, IL-6, IL-9, IL-10, IL-12 (p40), IL-12 (p70), IL-13, IL-17𝜶, eotaxin, G-CSF, GM-CSF, IFN-𝜸, KC, MCP-1 (MCAF), MIP-1𝜶, MIP-1β, RANTES and TNF-𝜶. The samples were diluted 1:4, and cytokine concentrations were derived by interpolating the measured fluorescence intensities to standard curves and correcting them using the corresponding dilution factor. The concentration of all cytokines (except IL-2 and IL-5) was calculated by Bio-Plex Manager software.

**Western Blotting**

Colon: Colons were collected from DSS and control mice and used for protein extraction. The tissues were homogenized at 4°C in lysis buffer (50 mM Tris-HCl pH 7.5, 150 mM NaCl, 5 mM EDTA, 0.5% Triton X-100, and protease-phosphatase inhibitors cocktail 1:100; Cell Signaling), sonicated at 30 KHz and centrifuged at 16200 xg for 5 minutes at 4°C. Protein content was quantified through the colorimetric Bradford assay (Sigma-Aldrich). 20 μg protein was loaded on a 12% polyacrylamide-SDS (SDS-PAGE) gel and transferred to a PVDF membrane (Merck). Membrane was blocked with a 5% w/v solution of bovine serum albumin (BSA) in Tris-Buffered Saline 0.1% Tween (TBS-T) for one hour at room temperature, then incubated overnight with anti-actin, and anti-Aqp4 diluted 1:1000 in 5% w/v BSA in TBS-T 1X at 4°C. After incubation with the anti-mouse secondary horseradish peroxidase (HRP)-conjugated antibody (diluted 1:3000 in 5% w/v BSA in TBS-T 1X) for 1 hour at room temperature, immunodetection was performed using the Azure Imager Biosystem instrument.

Brain tissues: Cortical and hippocampal frozen samples from control and DSS mice were lysed in modified RIPA buffer (10 mM Tris, pH 7.4, 150 mM NaCl, 1 mM EDTA, 0.1% SDS, 1% Triton X-100, protease inhibitors), sonicated, and centrifuged at 20,000 g for 10 min at 4 °C and the supernatant was kept for the immunoblot analysis. The protein content was quantified by using the colorimetric Pierce™ BCA Protein Assay Kit (Thermo Scientific, Cat# 23225, Waltham, MA, USA).

Samples were boiled for 5 min at 95 °C in SDS-PAGE sample buffer, separated by SDS-4-10% PAGE (30 µg/lane) and blotted onto PVDF membrane. Membranes were blocked for 1 h at room temperature in Tris-buffered saline-Tween (t-TBS: 20 mM Tris, pH 7.4, 150 mM NaCl, and 0.05% Tween 20), containing 5% (w/v) non-fat dried milk, and then probed with the following primary antibodies overnight at 4 °C: rabbit anti-CD45; rabbit anti-AQP4; mouse anti-Synaptophysin; rabbit anti-GABA Transporter 1 / GAT 1; mouse anti-Vimentin; rabbit anti-CD11b; mouse anti-excitatory amino acid transporter 1 (GLAST); mouse anti-excitatory amino acid transporter 2 (GLT-1); rabbit anti-postsynaptic density protein 95 (PSD95); mouse anti-glial fibrillary acidic protein (GFAP); rabbit anti β-actin.

After washes in t-TBS, membranes were incubated for 1 h at room temperature with the proper horseradish peroxidase-linked secondary antibodies (1:10000; anti-mouse (Sigma-Aldrich Cat# A9044, RRID: AB_258431); anti-rabbit (Sigma-Aldrich Cat# A9169, RRID:AB_258434). Immunoblots were visualized with an enhanced chemiluminescence Western blotting detection system Immobilon Forte Western HRP substrate (Sigma-Merck, Cat# WBLUF0500, Darmstadt, Germany). Images were acquired using the Alliance LD6 images capture system (Uvitec, Cambridge, UK) and analyzed with UVI-1D software (Uvitec).

**Histological analysis and Immunohistochemistry (IHC)**

The distal colon (n=3/group) was fixed in 10% neutral-buffered formalin (HT501128; Sigma – Aldrich, Merck KGaA, Darmstadt, Germany) at room temperature, dehydrated through a series of graded alcohols, and embedded in paraffin wax. Tissue sections were cut to a thickness of 5 microns and stained with hematoxylin (#SLCQ1056, Sigma-Aldrich, Saint Louis, MO, USA), and eosin (#SLCP2819, Sigma-Aldrich, Saint Louis, MO, USA) (H&E) for histological analysis. Pathological assessment of the colon was conducted using a 3-point scoring system, which evaluated inflammatory cell infiltration and injury extent, and a 4-point scoring system for crypt damage. The scoring criteria were defined as follows: (0) none; (1) < 20%; (2) 20–50%; (3) 50–70%; and (4) > 70%. Scoring was conducted in a blinded manner, and images were captured with a Leica DMRB microscope (Cat. #501007 Leica Microsystems, Wetzlar, Germany).

**Behavioral test batteries**

We performed the following behavioral test batteries:

***Animal cohort 3***

**Sucrose Preference Test**

On day 7, corresponding to 48 hours of wash-out after DSS treatment, the sucrose preference test was carried out, according to *(9, 10)* in order to investigate anhedonia. Briefly, 48 hours before the test, animals were left to adapt to an 0.8% of sucrose solution to prevent neophobia. Every 12 hours the position of water and sucrose was switched to avoid place preference. During the test day, animals were allowed to drink the sucrose solution for 1 hour in their home cage, with food and water provided *ad libitum*. Sucrose preference was calculated as the percentage of sucrose consumed over the total amount of liquids drunk.

**Open Field Test**

See main text.

**Novel Object Recognition Test**

On day 8 and 9, the Novel Object Recognition test was performed, as previously reported *(11)*. Briefly, at day 9 the training trial was performed, in which animals were exposed for 10 minutes to two identical objects (named familiar) in the same arena used for the Open Field paradigm. 24 hours later, during the test phase, one familiar object was replaced by a novel one and animals were free to explore the two objects for 10 minutes. Subsequently, a blind experimenter scored the time spent exploring the two different object during the test phase and the discrimination index, considered as a measure of discriminatory capacity, was calculated as follow: (time spent exploring the novel object - time spent exploring the familiar object) / (time spent exploring the novel object + time spent exploring the familiar object).

**Tail Suspension Test**

On day 10, the tail suspension test was performed in order to evaluate depressive-like behavior, as previously described *(12)*. Animals were acclimatized to the room for 1 hour, then they were suspended to a bar by attaching their tails with an adhesive tape and the test was recorded for 6 minutes. The immobility time was then measured by a blind experimenter (in seconds).

***Animal cohort 4***

**Hole Board Test**

On day 7, corresponding to 48 hours of wash-out after DSS treatment, we performed the Hole Board task, according to *(13)*. Briefly, mice were placed in a dark squared arena, containing a suspended board with 16 round-shaped holes, distributed at equal distances. Each animal was free to explore the holes for 5 minutes and an automatic counter recorded the number of head dipping in the holes (named poking holes), considered as an indirect index of anxiety-like behavior.

**Splash Test**

During day 7, the splash test was conducted, as previously described *(14)*. Mice were sprayed with a solution containing 10% of sucrose and recorded in their home cages for 5 minutes. The grooming behavior prompted by the viscosity of the sucrose solution is commonly considered as a measure of selfcare. Thus, an experimenter, blind to animal treatments, scored the latency to the first grooming behavior (in seconds) and the time spent performing selfcare (in seconds) over the 5 minutes of the test.

**Elevated Plus Maze Test**

See main text.

**Passive Avoidance Task**

During day 9 and 10, the passive avoidance paradigm was carried out, as previously described *(15)*. Briefly, animals were acclimatized for 1 hour in a dark room, then each mouse was placed in the passive avoidance apparatus, consisting in a two-chambered box with an automatic guillotine door (Ugo Basile, Camerio, Varese, Italy). One chamber had a full illumination and the other chamber was completely dark with an electrified floor grid. The paradigm lasted two days. On the first day (training trial), the guillotine door was opened 30 seconds after that the mouse was placed in the illuminated chamber and, once the mouse entered in the dark chamber, a footshock was applied (0.8 mA for 2 seconds). 24 hours later, on the test day, the latency time to enter into the dark compartment (expressed in seconds) was collected, as a direct measure of recollection of the aversive experience. The cut-off time was set at 300 seconds.

**Supplementary references**

1. C. Delprete, R. Rimondini Giorgini, E. Lucarini, T. F. S. Bastiaanssen, D. Scicchitano, N. Interino, F. Formaggio, F. Uhlig, C. Ghelardini, N. P. Hyland, J. F. Cryan, R. Liguori, M. Candela, J. Fiori, S. Turroni, L. Di Cesare Mannelli, M. Caprini, Disruption of the microbiota-gut-brain axis is a defining characteristic of the α-Gal A (-/0) mouse model of Fabry disease. *Gut Microbes* **15**, 2256045 (2023).

2. L. Micheli, L. Di Cesare Mannelli, F. Del Bello, M. Giannella, A. Piergentili, W. Quaglia, D. Carrino, A. Pacini, C. Ghelardini, The Use of the Selective Imidazoline I1 Receptor Agonist Carbophenyline as a Strategy for Neuropathic Pain Relief: Preclinical Evaluation in a Mouse Model of Oxaliplatin-Induced Neurotoxicity. *Neurotherapeutics* **17**, 1005–1015 (2020).

3. S. W. Provencher, Estimation of metabolite concentrations from localized *in vivo* proton NMR spectra. *Magnetic Resonance in Med* **30**, 672–679 (1993).

4. J. F. A. Jansen, W. H. Backes, K. Nicolay, M. E. Kooi, ^1^ H MR Spectroscopy of the Brain: Absolute Quantification of Metabolites. *Radiology* **240**, 318–332 (2006).

5. R. Kreis, The trouble with quality filtering based on relative C ramér‐ R ao lower bounds. *Magnetic Resonance in Med* **75**, 15–18 (2016).

6. J. Near, A. D. Harris, C. Juchem, R. Kreis, M. Marjańska, G. Öz, J. Slotboom, M. Wilson, C. Gasparovic, Preprocessing, analysis and quantification in single‐voxel magnetic resonance spectroscopy: experts’ consensus recommendations. *NMR in Biomedicine* **34**, e4257 (2021).

7. J. Cox, N. Neuhauser, A. Michalski, R. A. Scheltema, J. V. Olsen, M. Mann, Andromeda: A Peptide Search Engine Integrated into the MaxQuant Environment. *J. Proteome Res.* **10**, 1794–1805 (2011).

8. Y. Perez-Riverol, J. Bai, C. Bandla, D. García-Seisdedos, S. Hewapathirana, S. Kamatchinathan, D. J. Kundu, A. Prakash, A. Frericks-Zipper, M. Eisenacher, M. Walzer, S. Wang, A. Brazma, J. A. Vizcaíno, The PRIDE database resources in 2022: a hub for mass spectrometry-based proteomics evidences. *Nucleic Acids Research* **50**, D543–D552 (2022).

9. A. Iemolo, M. Valenza, L. Tozier, C. M. Knapp, C. Kornetsky, L. Steardo, V. Sabino, P. Cottone, Withdrawal from chronic, intermittent access to a highly palatable food induces depressive-like behavior in compulsive eating rats. *Behavioural Pharmacology* **23**, 593–602 (2012).

10. Y.-W. Chen, P. V. Rada, B. P. Bützler, S. F. Leibowitz, B. G. Hoebel, Corticotropin-releasing factor in the nucleus accumbens shell induces swim depression, anxiety, and anhedonia along with changes in local dopamine/acetylcholine balance. *Neuroscience* **206**, 155–166 (2012).

11. E. Mhillaj, M. G. Morgese, P. Tucci, A. Furiano, L. Luongo, M. Bove, S. Maione, V. Cuomo, S. Schiavone, L. Trabace, Celecoxib Prevents Cognitive Impairment and Neuroinflammation in Soluble Amyloid β-treated Rats. *Neuroscience* **372**, 58–73 (2018).

12. M. G. Morgese, M. Bove, M. Francavilla, S. Schiavone, S. Dimonte, A. L. Colia, M. Bevilacqua, L. Trabace, P. Tucci, Sublingual AKBA Exerts Antidepressant Effects in the Aβ-Treated Mouse Model. *Biomolecules* **11**, 686 (2021).

13. H. Takeda, M. Tsuji, T. Matsumiya, Changes in head-dipping behavior in the hole-board test reflect the anxiogenic andror anxiolytic state in mice. (1998).

14. A. Lama, C. Pirozzi, C. Annunziata, M. G. Morgese, M. Senzacqua, I. Severi, A. Calignano, L. Trabace, A. Giordano, R. Meli, G. Mattace Raso, Palmitoylethanolamide counteracts brain fog improving depressive‐like behaviour in obese mice: Possible role of synaptic plasticity and neurogenesis. *British J Pharmacology* **178**, 845–859 (2021).

15. A. Eagle, H. Wang, A. Robison, Sensitive Assessment of Hippocampal Learning Using Temporally Dissociated Passive Avoidance Task. *BIO-PROTOCOL* **6** (2016), doi:10.21769/BioProtoc.1821.

**Supplementary figures and figure legends**


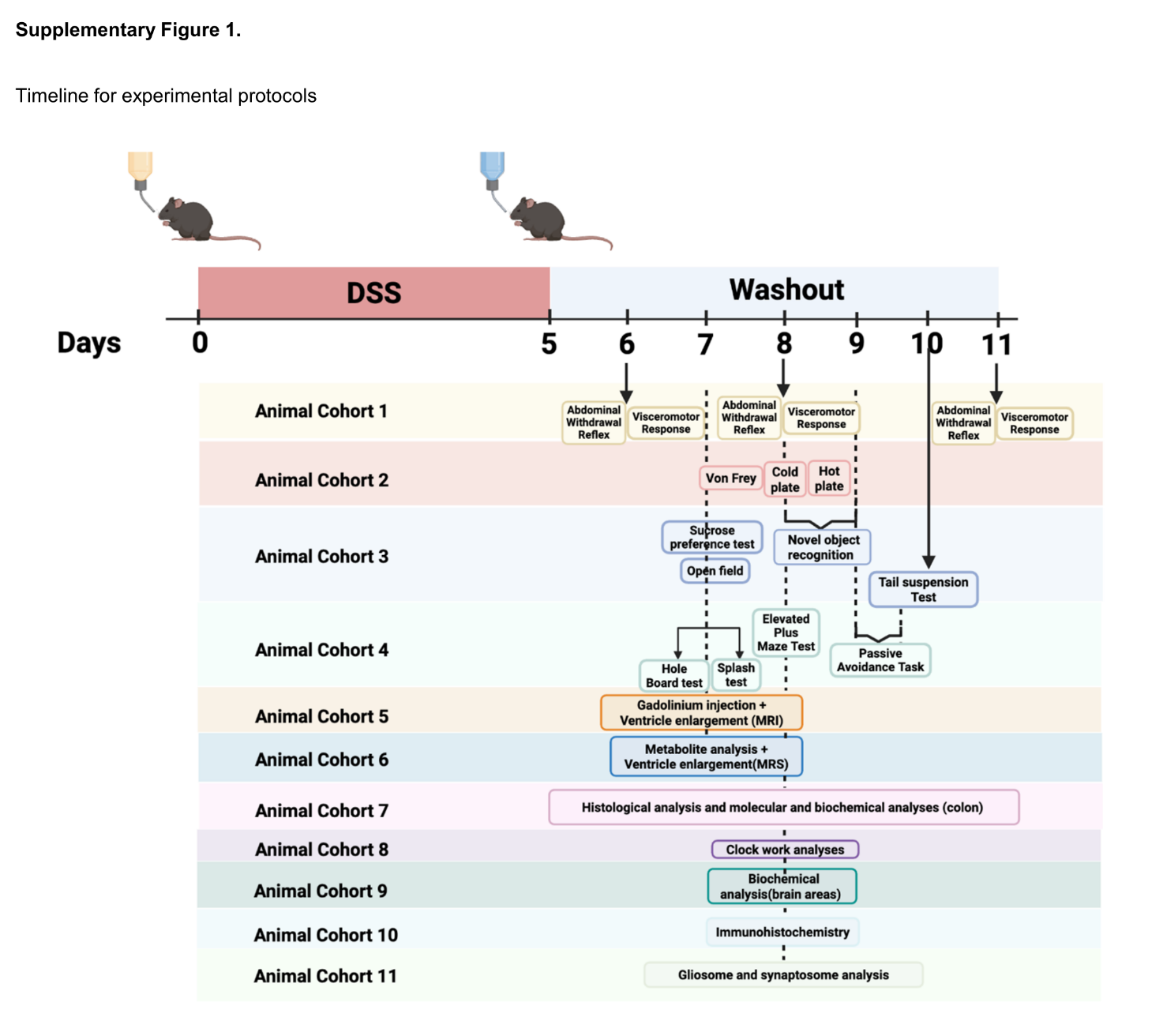


**Supplementary figure 1. Timeline of the different experimental settings**

**
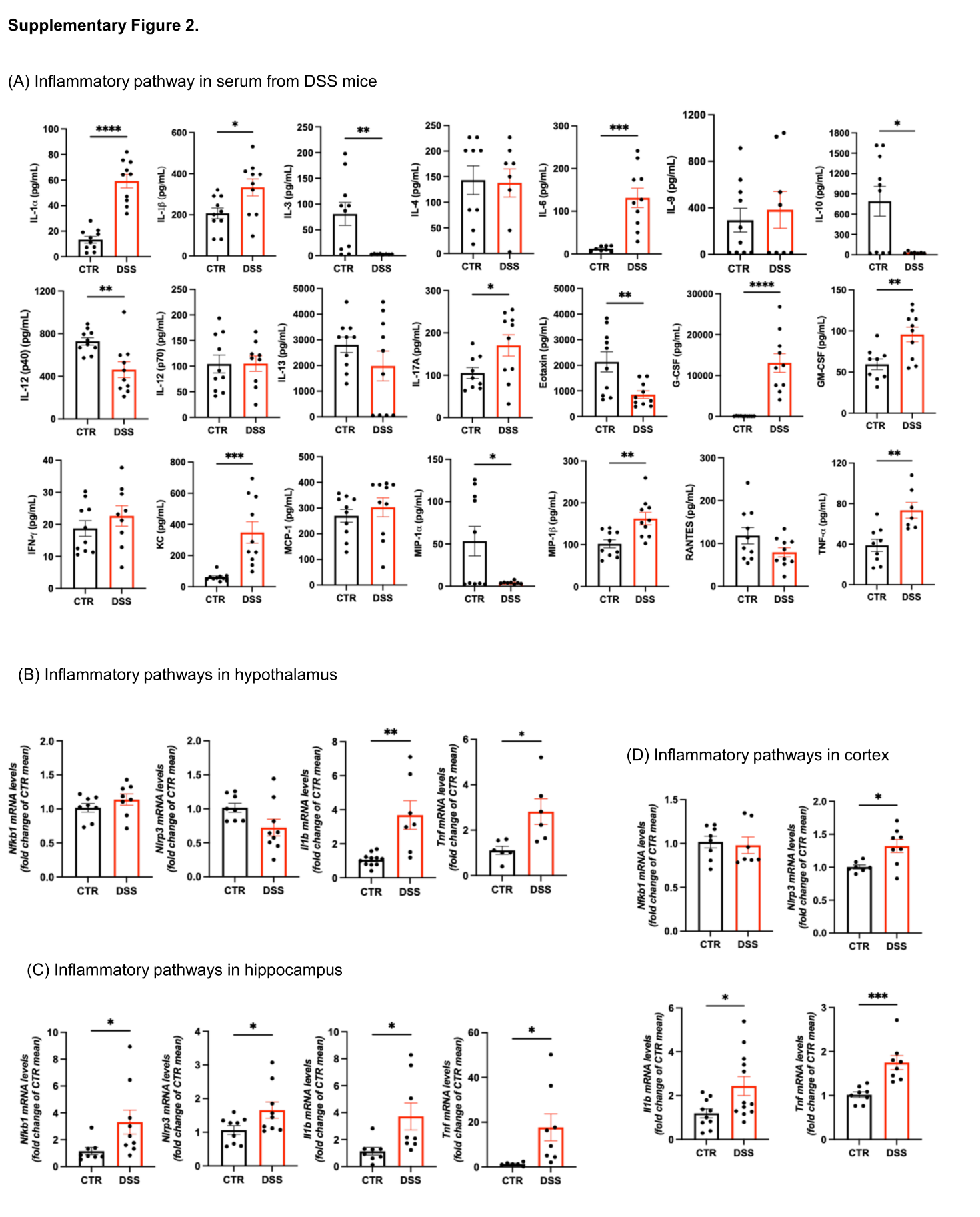
**

**Supplementary figure 2. Inflammatory pathways in DSS-treated mice.**

**A) Inflammatory pathway in serum from DSS mice.**The inflammatory and immunomodulatory markers IL-1𝜶, IL-1β, IL-3, IL-4, IL-6, IL-9, IL-10, IL-12 (p40), IL-12 (p70), IL-13, IL-17𝜶, eotaxin, G-CSF, GM-CSF, IFN-𝜸, KC, MCP-1 (MCAF), MIP-1𝜶, MIP-1β, RANTES and TNF-𝜶 were evaluated in serum from all groups by a 23-mouse cytokines Bio-Plex assay (n=10/each group, excluding cases where data were identified as outliers through the identify outliers analysis). Data were expressed as mean ± SEM and analyzed by unpaired two-tailed t-test (*p<0.05, **p<0.01, ***p<0.001, and ****p<0.0001 vs respective controls). Notably, we showed a reduction in serum IL-10 level, indicating that at the systemic level the inflammatory state predominates through the release of several pro-inflammatory cytokines, while at colonic level the increase of IL-10, an M2 cytokine, predisposes towards the recovery phase. Serum inflammatory data obtained via 23-mouse cytokines Bio-Plex assay were analyzed using the Identify Outliers test based on ROUT method (Q=1%) (GraphPad Software, San Diego, CA, USA; version 9.0). **(B-D) Inflammatory pathways in brain areas, hypothalamus (B), hippocampus (C), cortex (D).** The gene expression of the inflammatory markers Nfkb1, Nlrp3, Il1b, and Tnf was assessed in the hypothalamus, hippocampus, and cortex of CTR and DSS groups by Real-Time PCR (hypothalamus Nfkb1 n=8/each group, Nlrp3 CTR n=8 and DSS n=9, Il1b CTR n=11 and DSS n=7, Tnf n=6/each group; hippocampus Nfkb1 CTR n=8 and DSS n=9, Nlrp3 n=9/each group, Il1b n=8/each group, Tnf CTR n=6 and DSS n=8 and cortex Nfkb1 CTR n=8 and DSS n=7, Nlrp3 CTR n=7 and DSS n=8, Il1b CTR n=10 and DSS n=12, Tnf n=8/each group). Data are expressed by mean ± SEM and analyzed by unpaired two-tailed t-test (*p<0.05, **p<0.01, and ***p<0.001 vs respective controls).


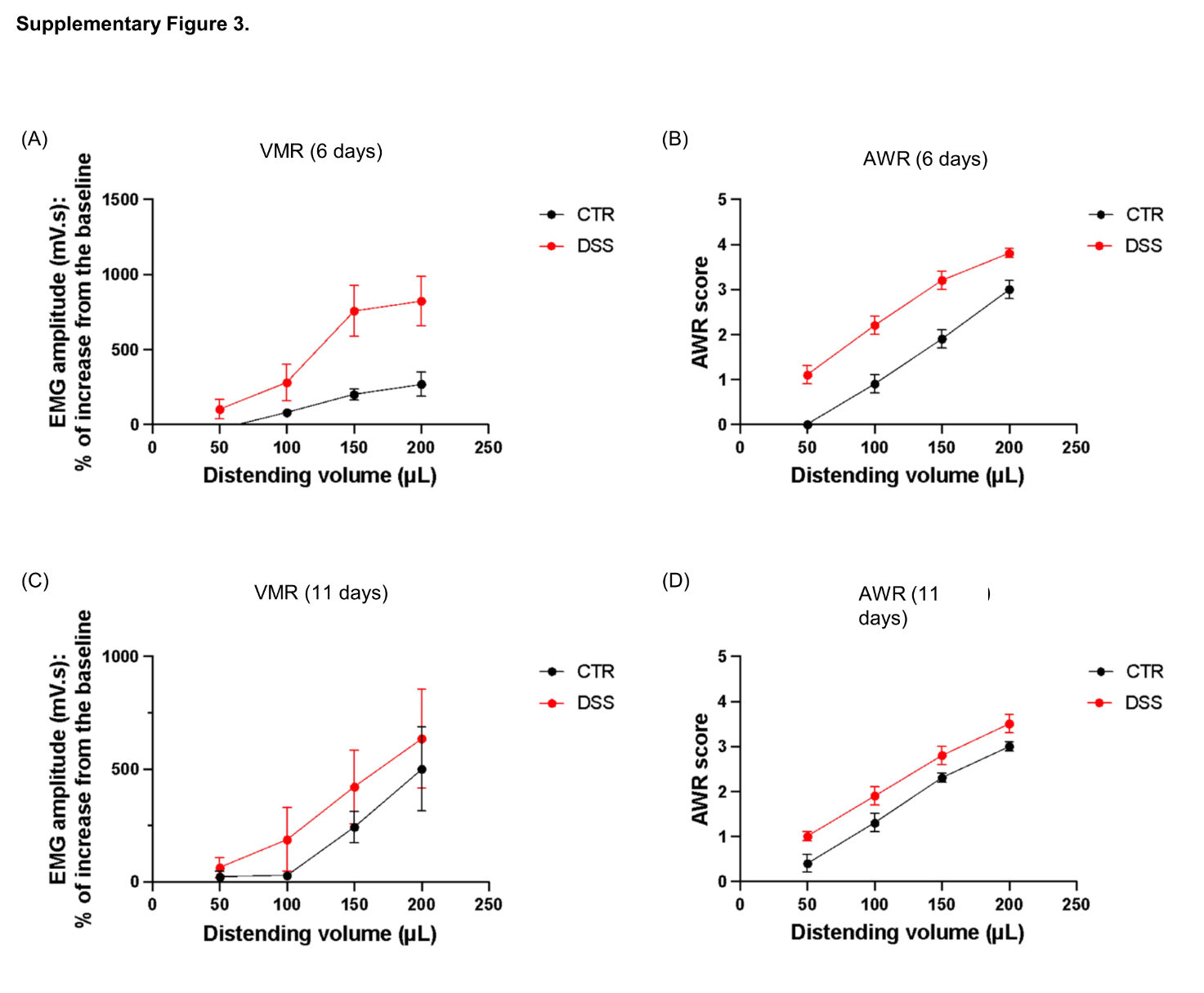


**Supplementary figure 3. Time course for visceral pain assessment.**

Preliminary studies were carried out to determine the optimal time point for further investigation into the relationship between gut dysfunction and brain alterations. Visceral pain assessments were performed 24h (A and B), 72h (figures 1Ga 1Gb) and 144h (C and D) after discontinuation of DSS treatment. Visceral sensitivity was assessed by measuring the electromyography (EMG) amplitude of abdominal contraction (VMR, viscero-motor response; A and C) and scoring behavioral responses (AWR, abdominal withdrawal reflex; B and D) in awake animals to colorectal distension with increasing volumes (50–200 µl balloon inflation). Twenty-four hours after the discontinuation of treatment, mice exposed to DSS displayed higher VMR than controls in response to colorectal balloon inflation with volumes ≥100 µl (A), while on day 8, DSS-treated mice showed a significantly higher VMR for all the stimuli applied to the colon (50-200 µl; 1Ga). On day 11 no significant difference in the VMR was observed between CTR and DSS-treated group, though the abdominal response continues to be slightly increased in post-colitis animals (C). Scoring the nocifensive behavior of animals to CRD through the AWR test, DSS-treated mice showed a significantly greater response than controls for all the stimuli applied to the colon (50-200 µl; B and 1Gb). On day 11, the difference in the AWR between CTR and DSS-treated mice was significant only at the lowest volumes (50-100 µl; D) of distension and its magnitude appeared reduced compared with day 8 (1Gb). Each value represents the mean ± SEM of 8-10 animals per group (24h, n = 9; 72h, n = 10; 144h, n = 8; for both the CTR and DSS group). *p<0.05 and **p<0.01 versus controls.


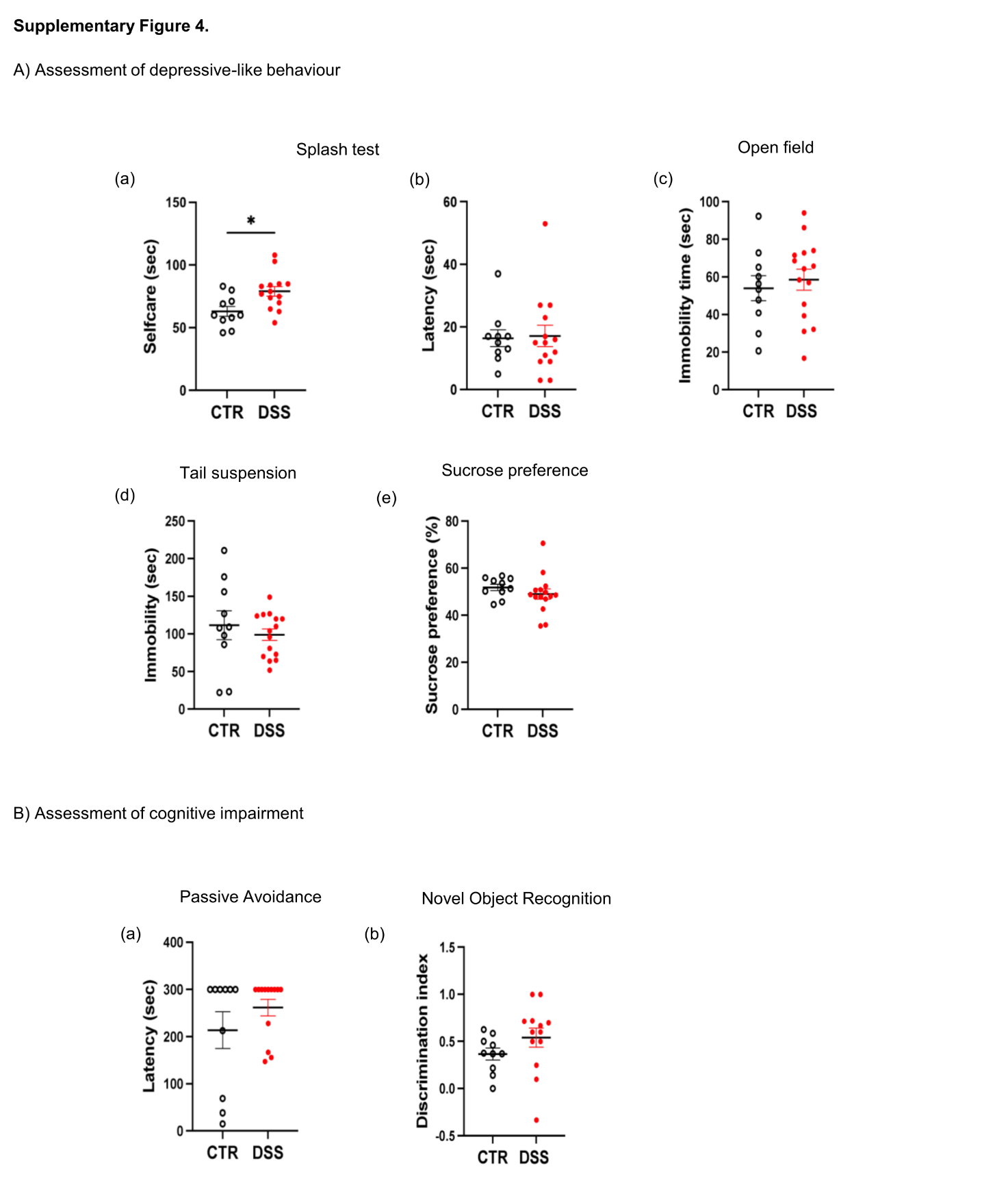


**Supplementary figure 4. Behavioral alterations in acute DSS-induced colitis mice.**

**A) Assessment of depressive-like behaviour**. (a) Latency to start selfcare behavior (in seconds) in the Splash test from control (CTR, black bar, n=10) and DSS (red bar, n=14) mice. Mann-Whitney test. Data are expressed as mean ± SEM. p=0.7402 DSS vs CTR. (b) Time spent performing self-care behavior (in seconds) in the Splash test from control (CTR, black bar, n=10) and DSS (red bar, n=14) mice. Data are expressed as mean ± SEM. Two-tailed Unpaired Student’s *t*-test, t=2,798, df=22, C.I.=4,123 to 27,73, η2=0.2625, p=0.0105; *p<0.05 DSS vs CTR. Concerning depressive-like behaviors, our results showed that DSS-treated mice did not report difference in the latency to start selfcare behavior in the Splash test, while it was reported report enhanced self-care duration compared to control animals. However, selfcare behavior is mainly characterized by a grooming component, thus, it cannot be ruled out that such result could be an expression of increased grooming activity, as already retrieved in the Open Field test. (c) Time spent performing immobility (in seconds) in the Open Field test from control (CTR, black bar, n=10) and DSS (red bar, n=15) mice. Data are expressed as mean ± SEM. Two-tailed Unpaired Student’s *t*-test, t=0,5295, df=23, C.I.=-13,42 to 22,65, η2=0.01205, p=0.6015 DSS vs CTR. (d) Time spent performing immobility (in seconds) in the Tail Suspension test from control (CTR, black bar, n=10) and DSS (red bar, n=15) mice. Data are expressed as mean ± SEM. Two-tailed Unpaired Student’s *t*-test with Welch’s correction, t=0,6248, df=11,87, C.I.=-57,79 to 32,06 η2=0,03186, p=0.5439 DSS vs CTR. In regard to immobility behavior, in both Open Field test and Tail Suspension test, there were no significant differences among the two experimental groups (c and d). (e) Sucrose preference (in percentage) in the Sucrose Preference test from control (CTR, black bar, n=10) and DSS (red bar, n=15) mice. Data are expressed as mean ± SEM. Two-tailed Unpaired Student’s *t*-test with Welch’s correction, t=1,116, df=21,89, C.I.=-8,098 to 2,432, η2=0.05384, p=0.2765 DSS vs CTR. When investigating anhedonia, DSS-treated mice did not show any differences in the percentage of sucrose preference in the Sucrose Preference test **(B) Assessment of cognitive impairment.** (a) Latency to enter the dark chamber (in seconds) during the test phase of the Passive Avoidance task from control (CTR, black bar, n=10) and DSS (red bar, n=13) mice. Mann-Whitney test. Data are expressed as mean ± SEM. p=0.4293 DSS vs CTR. (b) Discrimination index in the Novel Object recognition test from control (CTR, black bar, n=10) and DSS (red bar, n=13) mice. Data are expressed as mean ± SEM. Two-tailed Unpaired Student’s t-test, t=1,376, df=21, C.I.=-0,08964 to 0,440,3 η2=0.08271, p=0.1833 DSS vs CTR. DSS-treated mice did not present alterations in the discriminatory capacity, since no differences with controls were detected in the discrimination index measured in the Novel Object Recognition test (b). The same result was obtained in the Passive Avoidance task, in which DSS-treated animals did not show learning dysfunctions after an aversive experience, as shown in a, where no differences between DSS and control animals, in terms of latency to enter in the dark compartment after receiving an aversive stimulus, were reported.


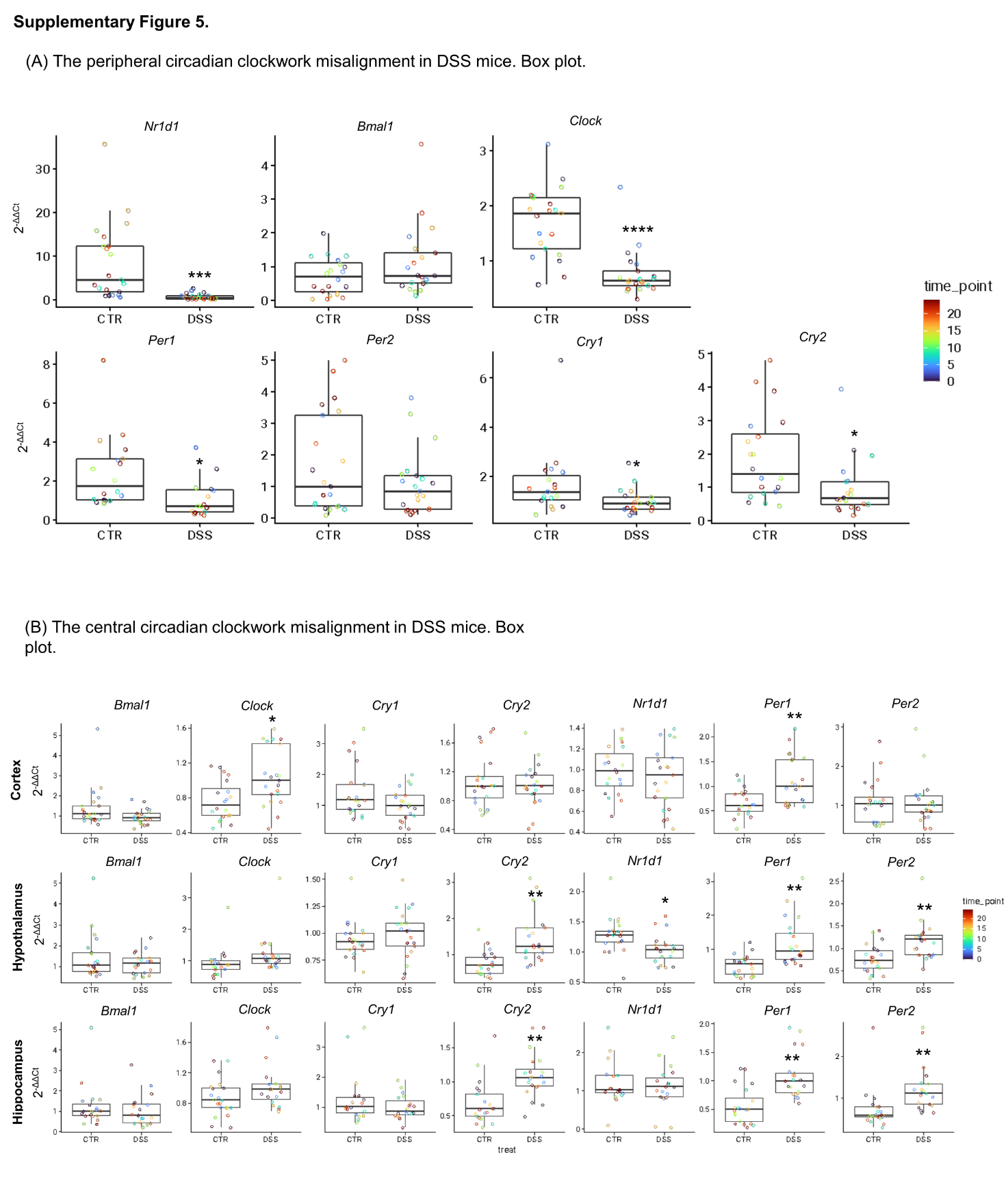


**Supplementary figure 5.**

**(A) The peripheral circadian clockwork misalignment in DSS mice. Box plot.** Gene expression (2^- ΔΔCt) of different clock genes across different time points (hours) in the colon dataset. The difference of gene expression (CTR used as reference) was modelled using a linear model with fixed effect exp~time_point+treat for each gene independently. `exp`, the outcome variable, corresponds to the estimated gene expression. `time_point` is the time points covariate and is treated as a fixed effect. `treat` is the main predictor under study, corresponding to the identity of the datapoint to either CTR or DSS group. Since each gene was tested independently, we also corrected the raw p value using the BH method (FDR). Genes with significant estimate for the `treat` covariate are labelled as follows: * FDR < 0.05, ** FDR <0.01, *** FDR<0.001, and **** FDR<0.0001. **(B) The central circadian clockwork misalignment in DSS mice. Box plot.** Gene expression (2^-ΔΔCt) of different clock genes across different time points (hours) in the hippocampus, hypothalamus, and cortex datasets. The difference of gene expression (CTR used as reference) was modeled using a linear model with fixed effect exp~time_point+treat for each gene independently. `exp`, the outcome variable, corresponds to the estimated gene expression. `time_point` is the time points covariate and is treated as a fixed effect. `treat` is the main predictor under study, corresponding to the identity of the datapoint to either CTR or DSS group. Since each gene was tested independently, we also corrected the raw p value using the BH method (FDR). Genes with significant estimate for the `treat` covariate are labeled as follows: * FDR < 0.05, ** FDR <0.01, *** FDR<0.001, and **** FDR<0.0001.
